# A set of nutrient limitations trigger yeast cell death in a nitrogen-dependent manner during wine alcoholic fermentation

**DOI:** 10.1371/journal.pone.0184838

**Published:** 2017-09-18

**Authors:** Camille Duc, Martine Pradal, Isabelle Sanchez, Jessica Noble, Catherine Tesnière, Bruno Blondin

**Affiliations:** 1 UMR SPO, INRA, Montpellier SupAgro, Université de Montpellier, Montpellier, France; 2 Lallemand SAS, Blagnac, France; University of Leicester, UNITED KINGDOM

## Abstract

Yeast cell death can occur during wine alcoholic fermentation. It is generally considered to result from ethanol stress that impacts membrane integrity. This cell death mainly occurs when grape musts processing reduces lipid availability, resulting in weaker membrane resistance to ethanol. However the mechanisms underlying cell death in these conditions remain unclear. We examined cell death occurrence considering yeast cells ability to elicit an appropriate response to a given nutrient limitation and thus survive starvation. We show here that a set of micronutrients (oleic acid, ergosterol, pantothenic acid and nicotinic acid) in low, growth-restricting concentrations trigger cell death in alcoholic fermentation when nitrogen level is high. We provide evidence that nitrogen signaling is involved in cell death and that either *SCH9* deletion or Tor inhibition prevent cell death in several types of micronutrient limitation. Under such limitations, yeast cells fail to acquire any stress resistance and are unable to store glycogen. Unexpectedly, transcriptome analyses did not reveal any major changes in stress genes expression, suggesting that post-transcriptional events critical for stress response were not triggered by micronutrient starvation. Our data point to the fact that yeast cell death results from yeast inability to trigger an appropriate stress response under some conditions of nutrient limitations most likely not encountered by yeast in the wild. Our conclusions provide a novel frame for considering both cell death and the management of nutrients during alcoholic fermentation.

## Introduction

During wine alcoholic fermentation, yeast cells have to withstand numerous stresses such as osmotic stress, low pH, high ethanol level and nutrient depletion. Some adaptation to these stresses is required to maintain high cell viability up to the end of alcoholic fermentation, allowing complete sugar consumption. Depending on the severity of the stress, yeast cells can lose their viability, which leads to sluggish or stuck fermentations [1]. Loss of viability during alcoholic fermentation is usually attributed to an insufficient availability of lipids, specifically sterols or unsaturated fatty acids, given that a membrane deficiency in these compounds is thought to alter cell resistance to ethanol [2].

Recent studies in yeast ageing have provided a novel framework to address the issue of cell death during fermentation. Actually, the loss of yeast viability in stationary phase can be addressed as chronological ageing since the survival of individual yeast cells in such non-dividing stationary phase can be used as a measure of the chronological lifespan [3]. An important conclusion from ageing studies is that yeast resistance to starvation can be influenced by the nature of the nutrient limiting cell growth [4]. Specifically, depending on nutrient limitation, yeast cells elicit different physiological responses and are unable to survive when starvation is due to an unusual nutrient limitation. During evolution, yeast has developed physiological responses to cope with nutrient limitations it frequently faced in natural environments, such as carbon, nitrogen, phosphate or sulfate (which will be hereafter described as usual limiting nutrients) but not for “unusual” limitations in some specific nutrients i.e. resulting from an induced auxotrophy (termed hereafter unusual limiting nutrients) [4]. Adaptation to starvation has been shown to involve nutrient sensing signaling pathways, i.e. target of rapamycin (TOR) and protein kinase A (PKA), their inactivation leading to enhanced chronological lifespan [5]. TOR pathway’s activity is modulated by the availability of nitrogen sources during starvation. In a recent work, Tesnière et al [6] have shown that, during alcoholic fermentation, the loss of yeast viability associated with yeast growth limitation by ergosterol was modulated by the medium’s nitrogen content, a high nitrogen level leading to enhanced cell death [6]. Consistent with a key role of TOR/Sch9 signaling in triggering cell death, both Tor inhibition by rapamycin and *SCH9* deletion resulted in increased cell survival. These data pointed to yeast inability to adapt to ergosterol limitation suggesting that it corresponds to an unusual nutrient limitation for yeast cells as previously defined. This is indeed consistent with yeast conditional requirement for ergosterol that restricts the auxotrophy to this compound to highly anaerobic conditions that are probably only encountered in industrial situations. We therefore examined whether other nutrients limitation could lead to a similar lack of adaptation to starvation and to a loss of cell viability during alcoholic fermentation. In addition to lipids, yeast is known to require several micronutrients such as thiamin, nicotinic acid, pantothenic acid, etc. to achieve alcoholic fermentation [7].

Little is known about the effect of growth limitation by these compounds on yeast viability during alcoholic fermentation. As nutrient disequilibriums leading to these situations are still not well-known and because the underlying mechanisms are poorly understood, the present paper aims at clarifying the molecular mechanisms involved in the occurrence of yeast cell death in response to nutritional unbalances through a combination of functional genomic approaches. We show here that several micronutrients limitations lead to cell death during alcoholic fermentations in a nitrogen-dependent manner. We characterized yeast response to starvation stress through a combination of functional approaches and show that cell death involves nitrogen signaling associated with an absence of stress response.

## Materials and methods

### Strains

We used the commercial wine yeast strain Lalvin EC1118^®^, a *Saccharomyces cerevisiae* strain isolated in Champagne (France) and manufactured by Lallemand (Montreal). The haploid strain 59a was obtained after sporulation of Lalvin EC1118^®^ as described by Ambroset et al [8]. A SCH9-deleted mutant of 59A was generated as described by Tesnière [6]. It was constructed by PCR-mediated gene disruption using the *loxP-KanMX- loxP* cassette of the pUG6 vector amplified with synthetic primers, to replace the ORF in 59A strain with a gene confering resistance to G418. Gene disruptions and constructs were confirmed by PCR.

### Synthetic culture media

Unless otherwise specified, a synthetic fermentation medium with 425 mg/L assimilable nitrogen (SM425) and 23% glucose + fructose (1/1), strictly buffered to pH 3.3 and simulating one third nitrogen and amino acid concentrations of a standard grape juice was routinely used [9]. This medium contained, per liter: 115 g glucose, 115 g fructose, 6 g citric acid, 6 g DL-malic acid, 750 mg KH2PO4, 500 mg K2SO4, 250 mg MgSO4.7H2O,155 mg CaCl2.2H2O, 200 mg NaCl, 4 mg MnSO4.H2O, 4 mg ZnSO4. 7H2O, 1 mg CuSO4.5H2O, 1 mg KI, 0.4 mg CoCl2.6H2O, 1 mg H3BO3, 1 mg (NH4)6Mo7O24, 20 mg myo-inositol, 2 mg nicotinic acid, 1.5 mg calcium pantothenate, 0.25 mg thiamine- HCl, 0.25 mg pyridoxine and 0.003 mg biotin. It also contained ammoniacal nitrogen and amino acids as nitrogen sources (per liter): 460 mg NH4Cl, 612 mg L-proline, 505 mg L-glutamine, 179 mg L-tryptophane, 145 mg L-alanine, 120 mg L-glutamic acid, 78 mg L-serine, 76 mg L-threonine, 48 mg L-leucine, 45 mg L-aspartic acid, 45 mg L-valine, 38 mg L-phenylalanine, 374 mg L-arginine, 33 mg L-histidine, 33 mg L-isoleucine, 31 mg L-methionine, 18 mg L-glycine, 17 mg L-lysine, 18 mg L-tyrosine and 13 mg L-cysteine. The medium was heat-sterilized (100°C, 10 min). Lipid factors (LF) were added to the fermentation medium after sterilization, to a final concentration of 530 mg/L oleic acid and 15 mg/L ergosterol. To evaluate the adaptation to nutrient limitations, eight main fermentations conditions were established with different concentrations in the culture medium of: ergosterol at 1.5 mg/L (Erg-: low ergosterol conditions), oleic acid at 18 mg/L (brought as Tween 80^®^, which is mainly a mix of oleate esters [10] (Ole-: low oleic acid conditions), pantothenic acid at 0.02 mg/L (Pan-: low pantothenic acid conditions) and nicotinic acid at 0.08 mg/L) (Nic-: low nicotinic acid conditions) with high: N+ (425 mg/L of yeast assimilable nitrogen) or low: N- (71 mg/L of yeast assimilable nitrogen) nitrogen level. Thiamin, biotin and inositol starvation were also tested in condition of excess nitrogen with a concentration of respectively 15 μg/L, 0.06 μg/L and 0.2 mg/L.

### Fermentation conditions and kinetics

The yeast strain Lalvin EC1118^®^ used in this study was precultured for 24 h at 28°C in a nutrient medium containing Yeast Nitrogen Base (YNB) (6.7 g/L) without amino acids and glucose (20 g/L) in Erlenmeyer flasks. When tested, the Tor kinase inhibitor rapamycin was added at 20 nM. The fermentation medium was then inoculated at 10^6^ cell/mL from this preculture. Yeast cultures were carried out in fermenters (350 mL containing 300 mL or 1.2 L containing 1 L medium), with fermentation locks (CO_2_ bubbling outlets filled with water). Fermentation media were routinely de-aerated prior to inoculation by bubbling pure argon for 5 min. Filling conditions were controlled and fermentations were carried out under anaerobic and isothermal conditions (24°C for 1,2L fermenters and 28°C for 350 mL fermenters), with permanent stirring (300 rpm). The amount of CO_2_ released was calculated from automatic measurements for 1.2 L fermenter (taken every 20 min) or from manual measurements of fermenter weight for 350 mL fermenters [11]. For 1.2 L fermenters, the CO_2_ production rate was calculated by sliding-window second-order polynomial fitting on the last 11 measurements in a custom-developed Labview application.

### Cell population densities and cell viability determinations

In all the different experiments, cell populations were counted using a Beckman-Coulter electronic particle counter. Cell viability was determined by flow cytometry using a C6 cytometer (Accuri, BD Biosciences): propidium iodide (PI) (Calbiochem) was added to the cell suspension (5 μL of a 0.1 mg/mL solution), and the samples mixed by gentle shaking. PI is a fluorescent nucleic acid stain (excitation 488 nm, emission 575 nm) which cannot penetrate intact cell membranes. PI flow cytometry analysis was performed 15 min after staining. Fluorescence data for cells stained by PI were collected in channel FL3. Viability was determined as the percentage of intact and fragile cells among all cells [12].

### Experimental design for assessing the stress response under micronutrient starvations

**Fig 1:**
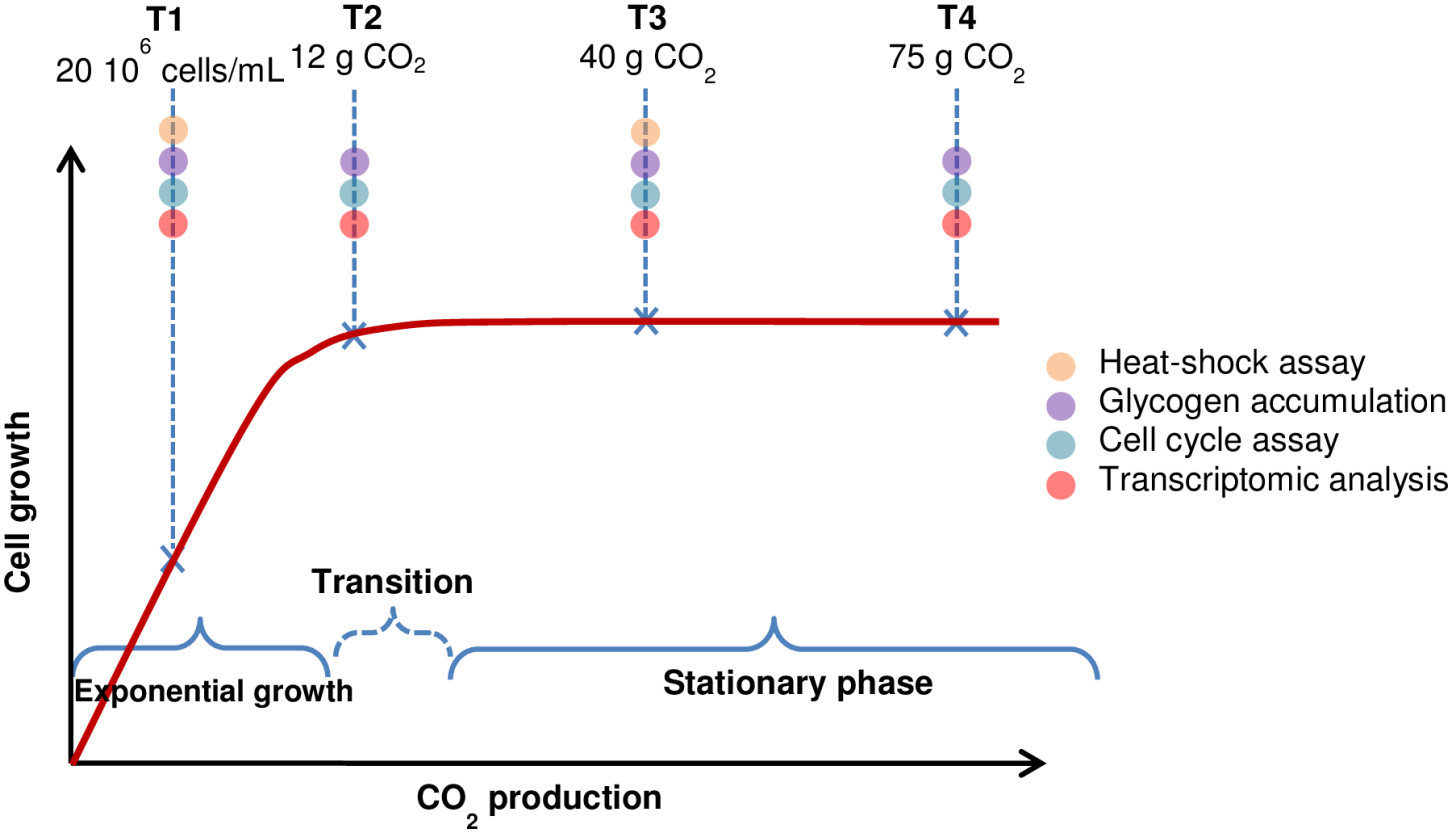
Representation of the different time-points at which experiments for assessing the stress response were performed.

For assessing stress response, we performed four experiments: a heat-shock sensitivity assay, a glycogen accumulation assay, a cell cycle assay and a transcriptomic analysis. We decided to perform it following a kinetic approach. We thus selected four time-points across fermentation T1, T2, T3 and T4 (Fig 1). For performing the heat-shock sensitivity assay, the glycogen accumulation or the cell cycle analysis, 10^7^ cells were harvested for each time-point. 10^9^ cells were needed for each time-point for performed the transcriptomic analysis. The first time-point T1 corresponds to a growth of 20 10^6^ cells/mL, during exponential growth (i.e. when no nutrient is limiting). The second time-point T2 was chosen at 12 g of CO_2_ produced which corresponds to the entry into stationary phase. The third and fourth time-points T3 and T4 were respectively taken at 40 g and 75 g of CO_2_ produced. T3 and T4 correspond to stationary phase.

We tested 6 nutrient conditions: two conditions that allow yeast cells to maintain their viability during wine fermentation and serve as controls, and 4 conditions of unusual nutrient starvation that lead to cell death.

The two conditions chosen allowing the maintenance of viability are a nitrogen starvation (N-) and a nitrogen plus ergosterol starvation (N-/Erg-) as we showed in the first part of the results that, in our conditions, yeast viability depends on nitrogen concentration availability. The other 4 conditions are those with excess of nitrogen and starved for oleic acid (N+/Ole-), ergosterol (N+/Erg-), pantothenic acid (N+/Pan-) or nicotinic acid (N+/Nic-). The times during the fermentation at which the samples were harvested are presented in S1 Table.

### Estimation of glycogen accumulation

For each sample, cells were washed with sterile water and stored at -80°C until used. 2.5 10^6^ cells were dropped on a nitro-cellulose membrane and fixed by vacuum using a Bio-Rad Apparatus. The membrane was exposed 2 min over iodine crystals and pictures directly taken as described by Enjalbert [13].

### Heat-shock sensitivity

For each sample, cells were heat-shocked at 50°C for 0, 5, 10 and 20 min. Five serial 10-fold dilutions were spotted onto YEPD plates and growth visualized after 2 days at 28°C as described by Klosinska et al [14].

### Cell cycle assay

Cell cycle measurements were done by flow cytometry using a C6 cytometer (Accuri, BD Biosciences) according to Delobel and Tesnière [15]. For each sample, cells were centrifuged, resuspended in 1 mL water and fixed in 8 ml 75% ethanol, added dropwise under continuous vortexing to avoid cell agglomeration. Samples were stored at least one night at 4°C. The samples were then centrifuged for 5 min at 2000 g and cell pellets resuspended in 1 mL PBS and centrifuged for 1 min at 13000 g at room temperature. The supernatant was discarded and enzymatic treatments were then performed to eliminate RNAs and proteins: the pellet was resuspended in 500 mL RNase A (2 mg.mL^-1^ in 10 mmol.L^-1^ Tris-Cl and 15 mmol.L^-1^ NaCl), incubated for 1 h at 37°C, and centrifuged; the pellet was resuspended in 200 mL proteinase K (1 mg.mL^-1^ in PBS), sonicated for 15 s in an ultrasonic bath (Branson Sonifier), incubated for 1 h at 50°C and centrifuged. The pellet was resuspended in 500 mL PBS and stored on ice until analysis. Before analysis, 50 μL of cells were incubated with SYTOX^®^ Green at 1 μmol.L^-1^ and directly observed using the flow cytometer. Fluorescence data for cells stained with SYTOX^®^ Green were collected in channel FL1.

### RNA extraction and microarray assay

Total RNAs were isolated from Lalvin EC1118^®^, at the selected time-points in conditions detailed previously, by the TRIzol^®^ method according to Chomczynski and Sacchi [16]. Aliquots of 10^9^ cells were harvested and quickly washed with 750 mL cooled (4°C) DEPC-treated water. Cells were pelleted and frozen in a -80°C methanol bath. Frozen cells were mechanically lysed through vortexing with glass beads (d = 0.3 mm) in 400 mL TRIzol^®^ (GIBCO BRL) at 4°C for 15 min. The liquid phase was collected and TRIzol^®^ added to a 4 mL final volume. The samples were mixed and incubated for 5 min at room temperature, and 800 mL chloroform was added. The mixture was then vortexed, incubated for 3 min and centrifuged (9,000 g for 15 min). The supernatant was centrifuged again (2,000 g for 2 min) in swing-out buckets. RNAs were pelleted from 2 ml aliquots of the supernatant by the addition of 2 mL cooled isopropanol (-20°C) and incubated for 10 min. The samples were centrifuged (9,000 g for 10 min) and the resulting nucleic acid pellet was washed twice with 750 mL 75% ethanol/DEPC-treated water and then dissolved in 150 μL of nuclease-free water (Qiagen). Total RNA from 100 μg aliquots of these preparations was purified with an RNeasy^®^ mini kit (Qiagen) following the RNA cleanup protocol, including membrane DNase digestion. RNAs were eluted with 2 x 30 μL of the provided RNAse-free water. RNA quality was verified through capillary electrophoresis using an RNA 6000 Nano LabChip Kit (Agilent Technologies). Samples of 100 ng purified RNA were labelled with Low input Quick Amp Labelling one-colour kit (Agilent Technologies) according to manufacturer’s recommendations (indirect labelling of mRNAs with Cyanin 3 dCTP dye). RNAs were hybridized on 8 x 15 k array Agilent standard Yeast V2 Gene Expression Microarrays (Agilent Technologies) for 17 h in a rotating oven at 65°C following manufacturer’s recommendations. A Genepix 4000B scanner was used for array digitalization: laser voltage was set to avoid signal saturation and data were extracted with GenePix^®^ Pro 7 software (Molecular Devices).

### Statistical analysis of microarray data

The R3.1.3 software was used for statistical analyses [17]. The raw microarray data were imported and normalized with the quantile method for normalization between arrays using the limma package [18]. On the normalized data set, we analyzed gene expression changes over time using the maSigPro package and the single series approach. maSigPro is a regression-based approach to identify genes with temporal expression changes [19]. For the first step of this method, we defined a binomial regression model for each gene expression over the 4 time-points:

$$\text{Y}={\text{b}}_{0}+{\text{b}}_{1}\text{time}+{\text{b}}_{2}{\text{time}}^{2}+\varepsilon$$

with:

- Y: normalized expression value
- time: the quantitative variable of the time points (min)
- b_0_: start value
- b_1_: the slope estimation (induction or repression of the gene)–linear effect
- b_2_: the shape estimation (first change in the temporal profile)–quadratic effect
- ε are independent N(0,σ^2^) error terms

We adjusted this model by the least-squared technique for each gene and only genes with significant changes over time (i.e. with an adjusted p-value threshold of 0.005 corrected by the Benjamini-Hochberg method) were selected. Then, a variable selection procedure was applied using stepwise regression to find significant coefficients for each gene (step.method = “backward”, alpha = 0.01). The list of differentially expressed genes according to the slope was generated and allowed us to define patterns of changes in expression over time (Rsquared cutoff = 0.8). These patterns were then clustered using a hierarchical classification analysis with the correlation distance and complete linkage method using cluster v3.0 and displayed with the JavaTreeView v1.1.5r2 [20,21].

For a functional analysis of the defined clusters, each cluster was analyzed using the web-based tool Funspec (http://funspec.med.utoronto.ca/; adjusted p.value = 0.05 with a Bonferroni correction method) and genes were classified into functional categories, biological processes and protein cellular localizations using the GO database [22]. In order to obtain individual patterns for some stress (or stress-related) gene expressions (*HSP30*, *HSP12*, *TPS1*, *HSP26*, *HSP104*, *SOD2*, *MSN2*, *MSN4*), the corresponding data were extracted from the transcriptomic analysis.

The complete microarray data set was deposited in the Gene Expression Omnibus (GEO) public repository (accession number GSE95152). Microarray description is under GEO accession number GPL17690.

## Results

### Identification of micronutrient limitations leading to yeast cell death during alcoholic fermentation

**Fig 2:**
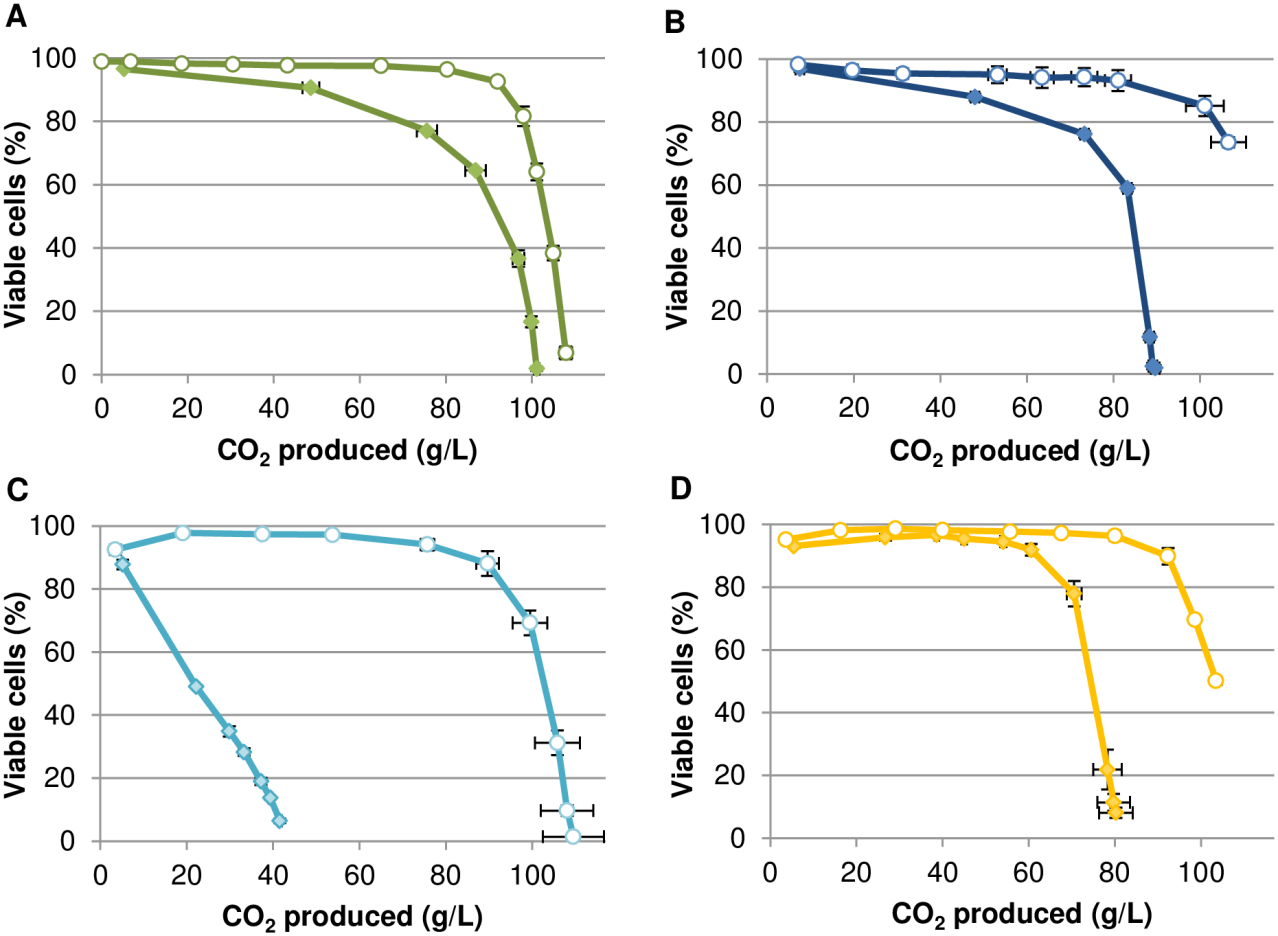
Impact of micronutrient starvations and nitrogen concentration on cell viability during alcoholic fermentation. (A) (green curves) Ole-: Oleic acid starvation (18 mg/L), (B) (dark blue curves) Erg-: ergosterol starvation (1.5 mg/L), (C) (light blue curves) Pan-: pantothenic acid starvation (0.02 mg/L) and (D) (yellow curves) Nic-: nicotinic acid starvation (0.08 mg/L). Open circles indicate N-: low nitrogen (71 mg/L YAN); full diamonds indicate N+: high nitrogen (425 mg/L YAN). Fermentations were performed in duplicate, error bars correspond to standard deviation.

In order to assess whether micronutrient limitations could trigger yeast cell death during alcoholic fermentation, strain Lalvin EC1118^®^ was set to ferment in a synthetic fermentation medium SM425 (containing 425 mg/L of yeast assimilable nitrogen) that mimics a grape must with various micronutrient limitations. The micronutrient concentrations were initially defined in order to obtain a loss of viability of at least 50% during the fermentation. We based our selection on preliminary work and a previous study [6]. In such media, all components were in excess except the selected micronutrient, which was set at a limiting concentration and thus was the first nutrient depleted. We examined the response to limitations in ergosterol and oleic acid (given the yeast cell requirements for these two compounds in anaerobiosis), thiamin, biotin, inositol, pantothenic acid and nicotinic acid. In the conditions used, yeast growth was restricted by the availability of the micronutrient (lipid or vitamin) while nitrogen was in excess. The variations in yeast cell viability throughout alcoholic fermentation are displayed in Fig 2 (for oleic acid, ergosterol, pantothenic acid and nicotinic acid) and S1 Fig (for thiamin, biotin and inositol). Since fermentation rates were highly variable among the situations examined and given the impact of ethanol on cell viability, cell viability is displayed in relation to the fermentation progress (amount of CO_2_ released) rather than in relation to time.

Oleic acid, ergosterol, pantothenic acid and nicotinic acid starvations led to cell death during fermentation in conditions of excess nitrogen. By contrast, thiamin, biotin and inositol starvations did not lead to cell death although they resulted in slow fermentations (S1 Fig). The rate of loss in cell viability differed depending on the micronutrient tested. Oleic acid and ergosterol starvations resulted in a loss of viability observable after 50 g of CO_2_ produced (Fig 2A & 2B) while cell death occurred after 60 g of CO_2_ produced when nicotinic acid was the limiting nutrient (Fig 2D). Starvation for pantothenic acid led to a loss of viability starting from the beginning of the fermentation until 40 g of CO_2_ produced which corresponds to the arrest of fermentation in this condition (Fig 2C). The three other micronutrients limitations also resulted in incomplete sugar fermentations (S2 Fig) but with higher CO_2_ production (Fig 2A, 2B and 2D).

The impact of assimilable nitrogen level on cell death was examined. A low level of nitrogen (71 mg/L) was defined so that it allowed a growth pattern similar to that obtained in musts limited by micronutrients and so that cells were starved simultaneously for the given micronutrient and nitrogen (S3 Fig). In such conditions, the biomass formed is equivalent in all situations so that the amount of micronutrient available per cell is similar independently of the nitrogen status.

When low nitrogen concentrations were used, cells retained high viability up to the end of the alcoholic fermentations whatever the micronutrient starvation. Thus, the amount of assimilable nitrogen modulates the yeast cell death associated with micronutrient limitations. These results indicate that the residual nitrogen sources are involved in triggering cell death when high nitrogen levels are used. Indeed analyses of nitrogen sources in fermentation media indicate that no residual amino acids or ammonium were available in nitrogen-limited fermentations while several nitrogen sources were still present in micronutrient-limited ones (S4 Fig).

### Assessing the role of nitrogen signaling in the control of the yeast cell death associated to micronutrient starvations

**Fig 3:**
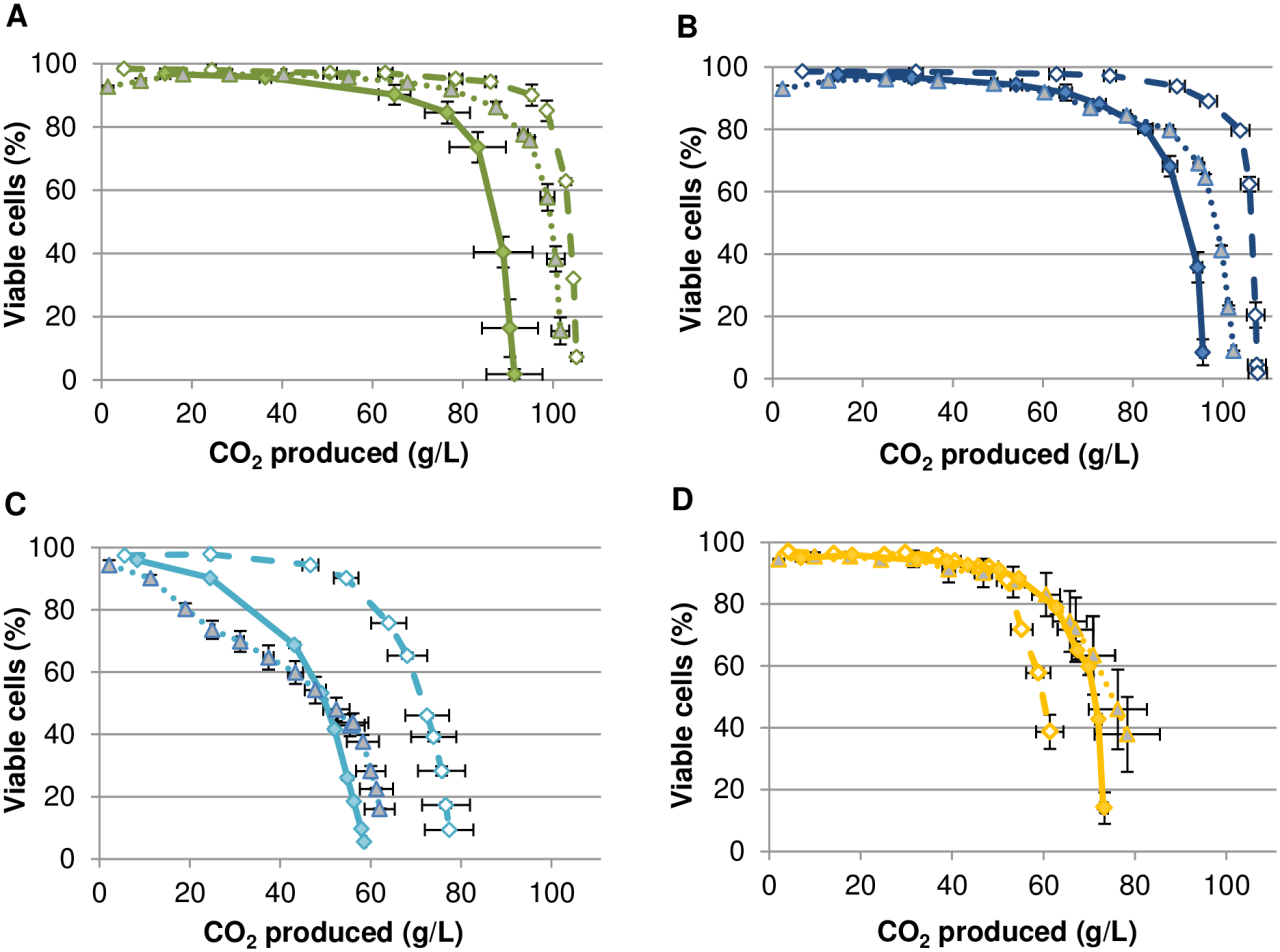
Effect of rapamycin and *SCH9* deletion on the *S*. *cerevisiae* 59A cell viability during alcoholic fermentation. (A) (green curves) Ole-: Oleic acid starvation (18 mg/L), (B) (dark blue curves) Erg-: ergosterol starvation (1.5 mg/L), (C) (light blue curves) Pan-: pantothenic acid starvation (0.01 mg/L) and (D) (yellow curves) Nic-: nicotinic acid starvation (0.02 mg/L). Full diamonds indicate fermentations performed using 59A. Open diamonds indicate fermentations performed using 59AΔSch9. Triangles indicate fermentation performed with rapamycin addition (20 nM). Fermentations were performed in duplicate using a SM142 (142 mg/l YAN), error bars correspond to the standard deviation.

Since nitrogen availability appeared to play a critical role in the triggering of yeast cell death, we examined the role of the Tor signaling pathway and of the downstream kinase Sch9 (Fig 3). The impact of the Tor kinase inhibitor rapamycin on cell death was examined using strain 59A (a haploid derivative of Lalvin EC1118^®^) and the selected set of micronutrient starvations.

The addition of rapamycin led to increased cell viability in fermentations limited for oleic acid or ergosterol (Fig 3A & 3B). In the ergosterol-limited medium, the rapamycin treatment led to a fermentation arrest at 102 g of CO_2_ produced whereas it stopped at 96 g of CO_2_ produced for the non-supplemented condition. Rapamycin treatment did not allow a full recovery of cell viability towards the end of the fermentation. For pantothenic acid starvation, arrests of fermentation were observed at 59 and 62 g of CO_2_ produced respectively for non-supplemented and rapamycin supplementation conditions. For nicotinic acid starvation, arrest of fermentation was observed at 73 and 78 g of CO_2_ produced respectively for non-supplemented and rapamycin supplementation conditions. For viability, standard deviations do not allow to differentiate the effect of a rapamycin treatment (Fig 3C & 3D). On the contrary, rapamycin enhanced cell death at the beginning of the pantothenic-limited fermentation, suggesting some toxic effect under this micronutrient limitation (Fig 3C). By contrast, deletion of the Tor target gene *SCH9* restored high yeast viability in oleic acid, ergosterol and pantothenic acid starved fermentations. However, no effect was observed in the nicotinic acid limited fermentation (Fig 3D). These results are consistent with the involvement of Tor/Sch9 signaling in cell death when yeast cells are starved for oleic acid and ergosterol. They are ambiguous concerning their involvement when cells are starved for pantothenic acid since they show that Sch9 is involved but that Tor inhibition had no effect. Unexpectedly, these data strongly suggest that cell death associated with nicotinic starvation does not involve Tor/Sch9 signaling.

### Micronutrient limitations fail to trigger phenotypic stress resistance

**Fig 4:**
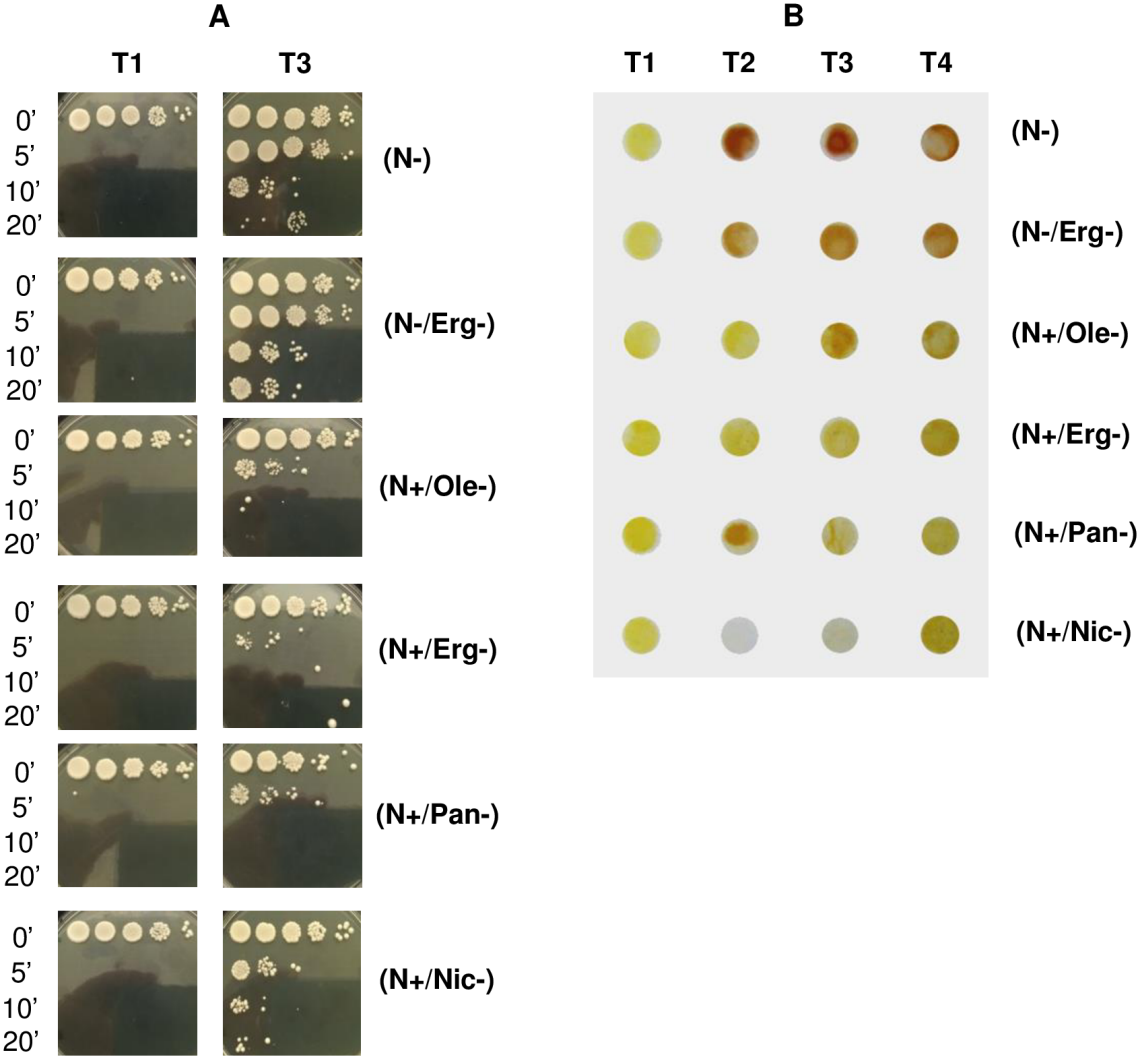
Assessment of stress resistance triggering during alcoholic fermentation. By (A) heat shock sensitivity assay and (B) visualization of glycogen storage for N-: low nitrogen, 71 mg/L YAN; N-/Erg-: low nitrogen/low ergosterol, 71 mg/L YAN, 1.5 mg/L ergosterol; N+/Ole-: high nitrogen/ low oleic acid, 425 mg/L YAN, 18 mg/L oleic acid; N+/Erg-: high nitrogen/ low ergosterol, 425 mg/L YAN, 1.5 mg/L ergosterol; N+/Pan-: high nitrogen / low pantothenic acid, 425 mg/L YAN, 0.02 mg/L pantothenic acid and N+/Nic-: high nitrogen/ low nicotinic acid, 425 mg/L YAN, 0.08 mg/L nicotinic acid.

Since nutrient limitations led to variable ability to cope with fermentation stress and maintain yeast viability, we examined the impact of nutrients limitations on the triggering of stress responses. We selected a set of conditions maintaining high viability (N- and N-/Erg-) or leading to cell death as previously described (N+/Erg-, N+/Ole-, N+/Pan-, N+/Nic-). The patterns of cell viability during the corresponding fermentations are displayed in S5A Fig. The cell growth and fermentation rates are shown in S5B and S5C Fig. To address the triggering of the stress response in this set of conditions, we examined yeast ability to withstand a short heat-shock (50°C) at 2 fermentation time-points (see Materials & methods). The first point (T1) examined was during exponential growth (20 10^6^ cells/ml) during which yeast cells do not experience nutrient starvation and the second point (T3) in stationary phase (at 40 g of CO_2_ produced) (Fig 4A).

In exponential growth (T1, 20 10^6^ cells/mL), cells did not survive any heat-shock irrespective of conditions and limiting nutrient. This is consistent with an absence of stress response at this stage. In stationary phase (T3, 40 g of CO_2_ produced), cells under nitrogen limitation condition alone were able to withstand heat shock (up to 10 minutes). Moreover, yeast cells limited for both nitrogen and ergosterol (N-/Erg-) were able to withstand the 20-minute thermal stress, thus displaying higher stress resistance in this situation. By contrast, yeast in presence of high nitrogen but starved for oleic acid, ergosterol, pantothenic acid or nicotinic acid (N+/Erg-, N+/Ole-, N+/Pan-, N+/Nic-) were not able to survive any thermal treatment. Indeed, the ergosterol-limited condition yielded yeast cells highly sensitive to thermal treatments (they did not survive a 5-minute shock) while nicotinic acid-limited cells were slightly more resistant. These data indicate that, in our conditions, yeast ability to successfully cope with thermal stress is strongly dependent on the limiting nutrient. While nitrogen limited condition resulted in stress-resistant yeast cells, the other nutrient limitations addressed were not able to trigger an appropriate stress response.

Glycogen accumulation is considered to be a good indicator of yeast stress response [23]. Glycogen storage was monitored by iodine staining of yeast at 4 points of alcoholic fermentation (Fig 4B). In exponential growth (T1: 20. 10^6^ cells/ml) the glycogen content (revealed by a yellow tint of the yeasts) was low in all conditions whatever the nutrient limitation. In stationary phase (at T2: 12 g, T3: 40 and T4: 75 g of CO_2_ produced), yeasts starved for nitrogen (N- and N-/Erg-) displayed an increased content in glycogen, with a brown tint after iodine staining. However, under single micronutrient limitation for either oleic acid, ergosterol, pantothenic acid or nicotinic acid (N+/Erg-, N+/Ole-, N+/Pan-, N+/Nic-), yeast cells did not accumulate glycogen. These results are consistent with an absence of stress resistance triggering in stationary phase whenever cell growth is limited by any of the 4 micronutrients tested.

### Cell cycle analysis

**Fig 5:**
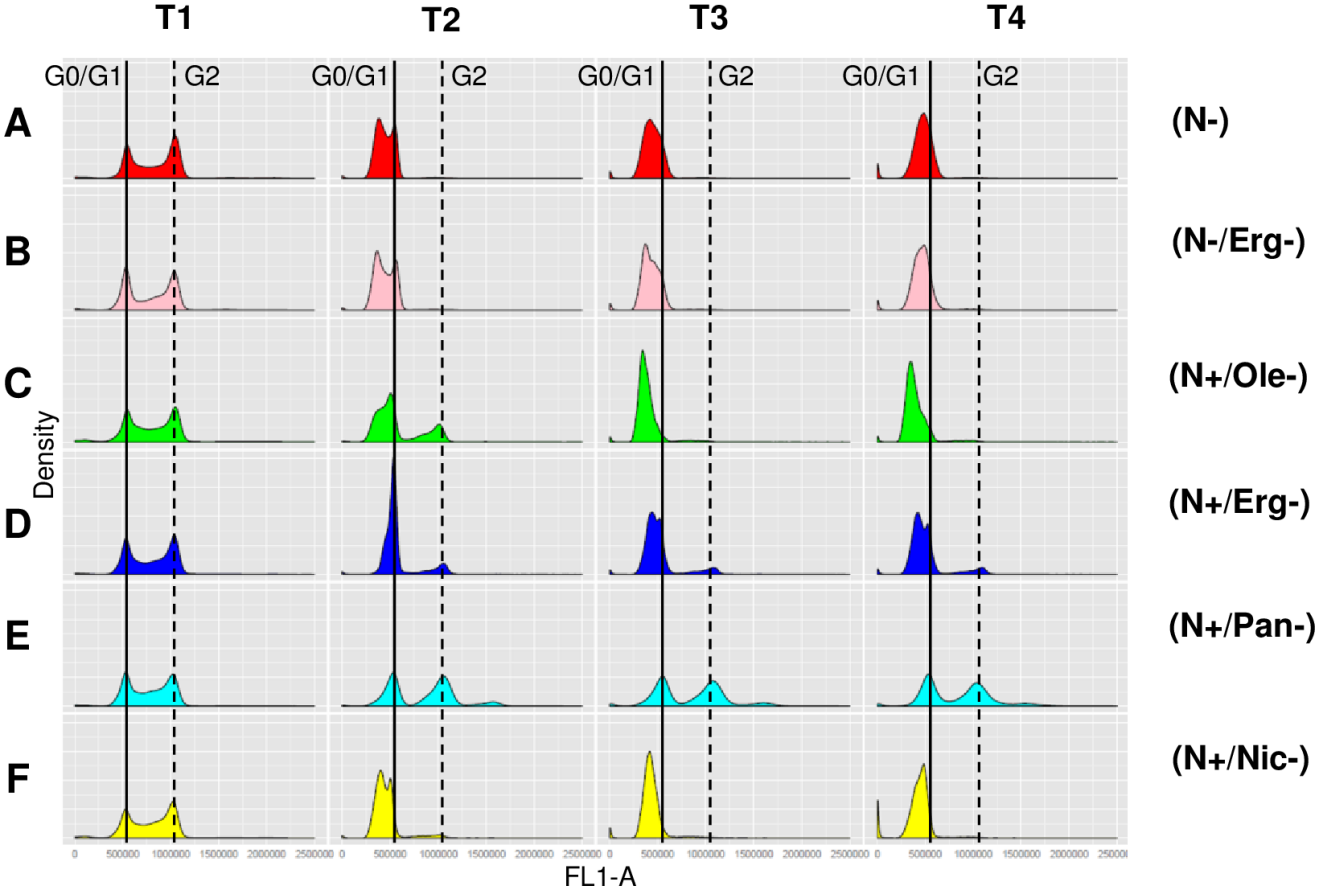
Cell cycle of yeast during alcoholic fermentation in the set of 6 nutrient conditions. Cells were collected at different times during alcoholic fermentation (T1, 20 10^6^ cells/mL; T2, 12 g CO_2_ produced; T3, 40 g CO_2_ produced; T4, 75 g CO_2_ produced) for: (A) N-: low nitrogen, 71 mg/L YAN; (B) N-/Erg-: low nitrogen/low ergosterol, 71 mg/L YAN, 1.5 mg/L ergosterol; (C) N+/Ole-: high nitrogen/ low oleic acid, 425 mg/L YAN, 18 mg/L oleic acid; (D) N+/Erg-: high nitrogen/ low ergosterol, 425 mg/L YAN, 1.5 mg/L ergosterol; (E) N+/Pan-: high nitrogen/ low pantothenic acid, 425 mg/L YAN, 0.02 mg/L pantothenic acid and (F) N+/Nic-: high nitrogen/ low nicotinic acid, 425 mg/L YAN, 0.08 mg/L nicotinic acid. Curves show the mean of two biological replicates.

We assessed the ability of yeast cells to arrest their cell cycle in G0/G1 phase upon nutrient depletion under the various situations (Fig 5). In exponential growth, cells exhibited a similar DNA content profile in the different conditions, with G1 and G2 peaks consistent with cells in division. The two nitrogen-limited cultures exhibited a clear cell cycle arrest with cells in G0/G1 phase (Fig 5A & 5B). A similar behavior was observed for cells starved for nicotinic acid (N+/Nic-) (Fig 5F). Oleic acid starvation (N+/Ole-) led to a high amount of cells in G2 phase at 12 g of CO_2_ (Fig 5C) indicating that some of them were still dividing. In this condition, cells resumed a G0/G1 arrest at 40 g of CO_2_ produced. Actually, these cells exhibited a lower DNA fluorescence peak than cells at G0/G1; the origin of such low fluorescence is unclear although such results have been previously reported [24]. Ergosterol starvation led mainly to cells arrested at the G0/G1 phase with however a small fraction in G2 phase (Fig 5D). An even more pronounced blockade of the cell cycle occurred in the limitation in pantothenic acid with nearly half of the population arrested in G2 phase (Fig 5E). The analyses point to a heterogeneous behavior of cells in these situations with various perturbations of the patterns of cell cycle arrest.

### Changes in the yeast transcriptome in response to nutrient starvations

To get insights into the regulatory mechanisms associated with cell death during alcoholic fermentations, we monitored gene expression in Lalvin EC1118^®^ strain under the six selected nutrient conditions. In each condition, the first sampling for gene expression was performed during the growth phase while the 3 others corresponded to cells in stationary phase at different levels of fermentation progress (sugar consumption and ethanol amount were similar for each condition). A linear regression was used to select genes exhibiting a statistically significant dependence with time during fermentation (see Material and methods). Thus, 1946 regulated genes were hierarchically classified into 28 clusters with significant dependence on time for the 6 nutrients conditions tested.

**Fig 6:**
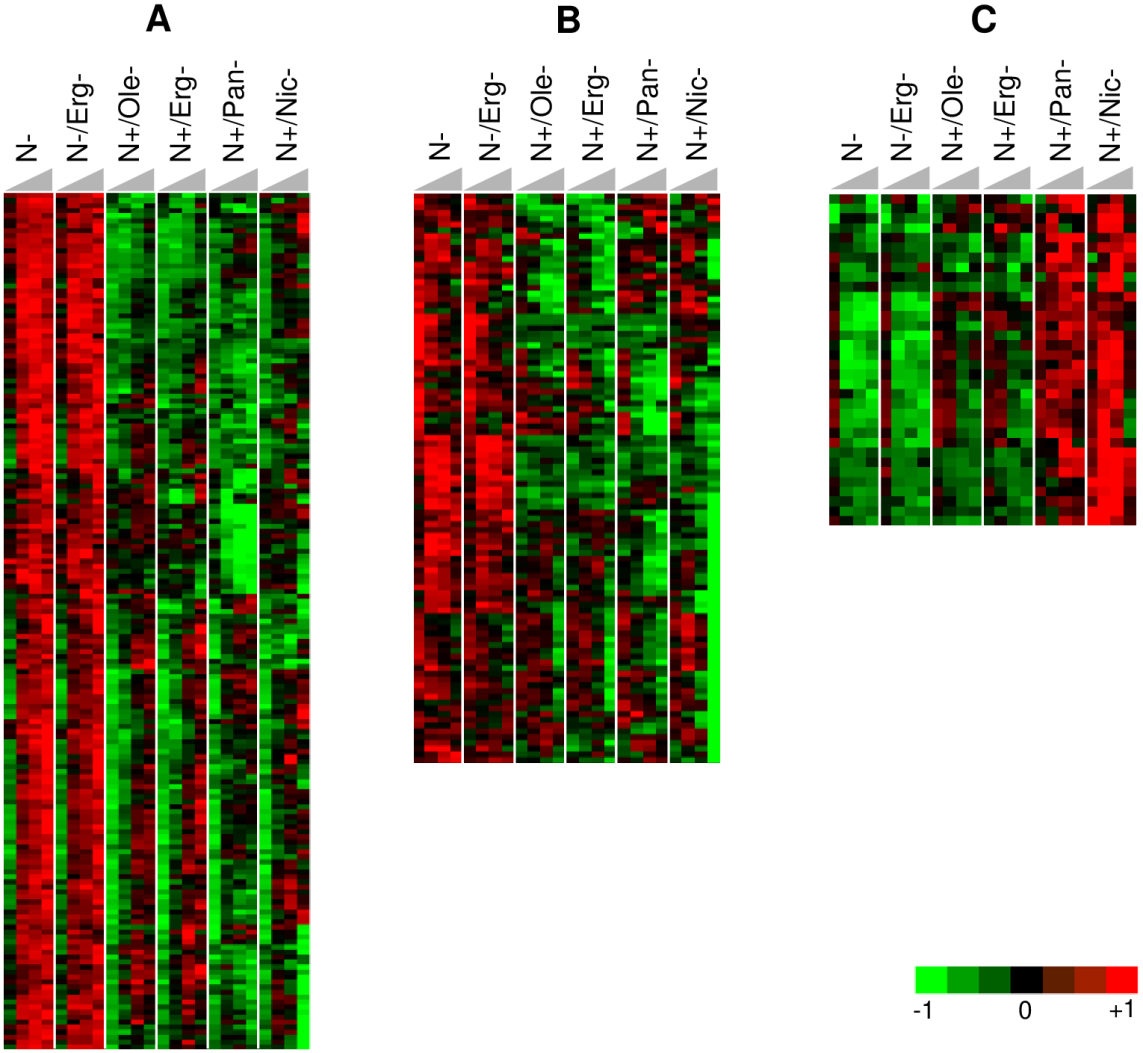
Genes expression with similar pattern during alcoholic fermentation. (A) Genes with low expression in micronutrient starvation compared with nitrogen starvations (cluster 10). Majors GO biological process enrichments: electron transport chain, tricarboxylic acid cycle, proline metabolic process. (B) Genes with low expression in micronutrient starvation compared with nitrogen starvation (cluster 24). Majors GO biological process enrichments: allantoin catabolic process, purine base metabolic process, amino acid transport, nitrogen compound metabolic process. (C) Highly expressed genes during micronutrient starvations compared with nitrogen starvations and genes specific to pantothenic acid and nicotinic acid starvation (cluster 14). Majors GO biological process enrichments: oxidation-reduction process, cellular amino acid biosynthetic process, cysteine biosynthetic process, lysine biosynthetic process. For: N-: low nitrogen, 71 mg/L YAN; N-/Erg-: low nitrogen/low ergosterol, 71 mg/L YAN, 1.5 mg/L ergosterol; N+/Ole-: high nitrogen/ low oleic acid, 425 mg/L YAN, 18 mg/L oleic acid; N+/Erg-: high nitrogen/ low ergosterol, 425 mg/L YAN, 1.5 mg/L ergosterol; N+/Pan-: high nitrogen/ low pantothenic acid, 425 mg/L YAN, 0.02 mg/L pantothenic acid and N+/Nic-: high nitrogen/ low nicotinic acid, 425 mg/L YAN, 0.08 mg/L nicotinic acid; transcriptomic assays were performed at four time points during alcoholic fermentation (T1, 20 10^6^ cells/mL; T2, 12 g CO_2_ produced; T3, 40 g CO_2_ produced; T4, 75 g CO_2_ produced) indicated by the grey triangle. Results show the mean of biological triplicates.

**Fig 7:**
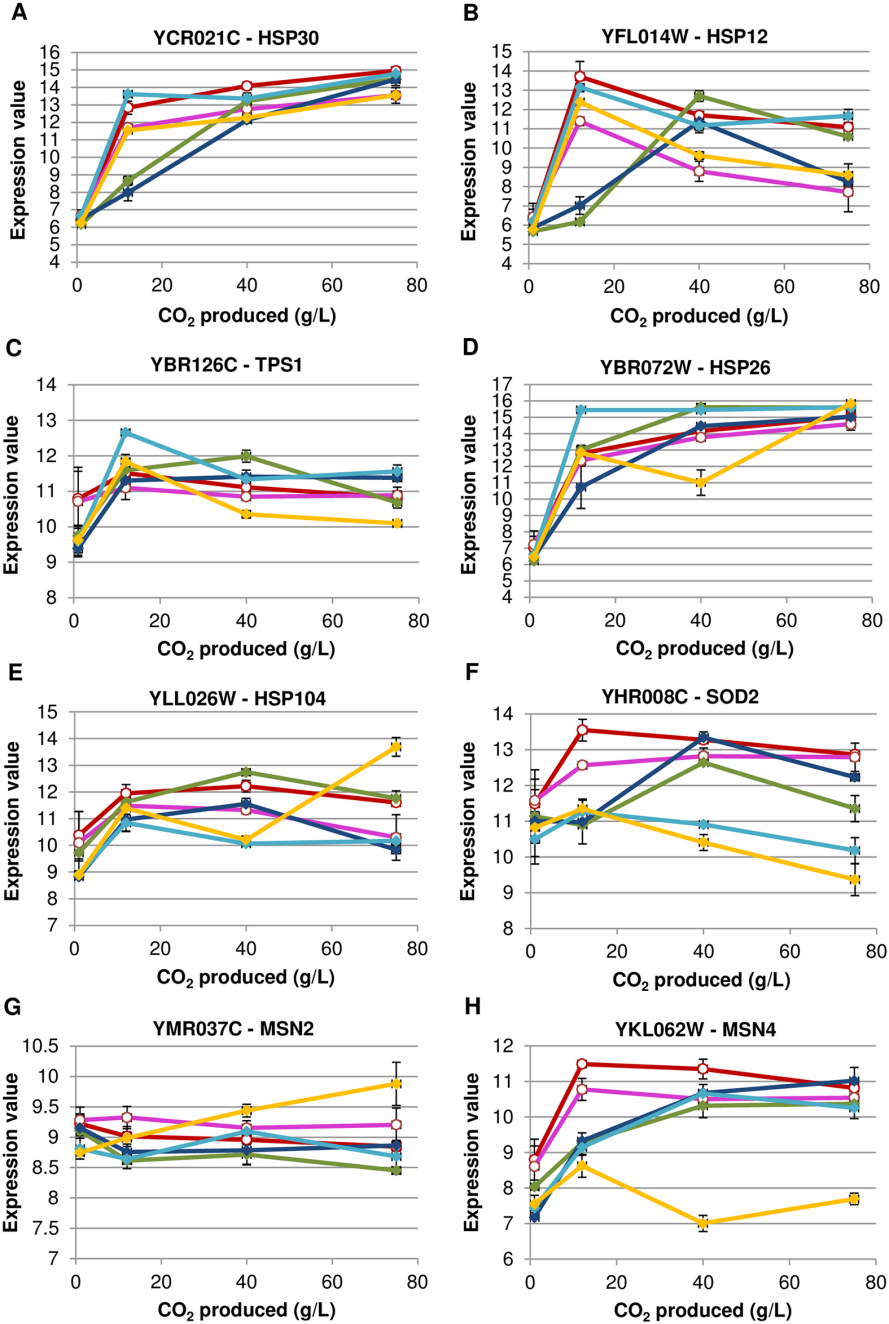
Expression pattern (arbitrary units) of several stress (or stress-related) genes during alcoholic fermentation under 6 nutrient starvation conditions. (A) *HSP30*, (B) *HSP12*, (C) *TPS1*, (D) *HSP26*, (E) *HSP104*, (F) *SOD2*, (G) *MSN2*, (H) *MSN4* for: (red curves) N-: low nitrogen (71 mg/L YAN); (pink curves) N-/Erg-: low nitrogen/ low ergosterol (71 mg/L YAN, 1.5 mg/L ergosterol); (green curves) N+/Ole-: high nitrogen/ low oleic acid (425 mg/L YAN, 18 mg/L oleic acid); (dark blue curves) N+/Erg-: high nitrogen/ low ergosterol (425 mg/L YAN, 1.5 mg/L ergosterol); (light blue curves) N+/Pan-: high nitrogen / low pantothenic acid (425 mg/L YAN, 0.02 mg/L pantothenic acid); and (yellow curves) N+/Nic-: high nitrogen/ low nicotinic acid (425 mg/L YAN, 0.08 mg/L nicotinic acid). Data were obained from the transcriptomic analysis. Curves show the mean of fermentations performed in triplicate, error bars correspond to the standard deviation.

We focused on clusters separating the conditions preserving viability from those leading to high cell death (Fig 6A & S6 Fig for details, Fig 6B & S7 Fig for details). We identified 2 clusters, i.e. cluster 10 with 166 genes and cluster 24 associating 98 genes, all exhibiting a strong induction brought by conditions of nutrient starvation supporting high viability (N- and N-/Erg-) but no induction in the four conditions leading to cell death. Cluster 10 is enriched in genes involved in mitochondrial respiration, electron transport processes, TCA cycle, membrane transport and detoxification. Cluster 24 contains genes involved in nitrogen catabolism, purine metabolism and membrane transport including nitrogen substrates. Many genes in both these clusters are under the control of the nitrogen catabolic repression (NCR), (*PUT*, *DAL* genes, *MEP2*, *PTR2*, *OPT* genes). Their differential expression is consistent with a depletion of nitrogen source in the two nitrogen-limited conditions and a release from NCR, while in the other conditions nitrogen sources prevent this regulation upon starvation. Surprisingly the *TOR1* and *SCH9* genes are members of this cluster with a higher expression in the two nitrogen-starved conditions, though their signaling is expected to decrease. This is thus consistent with a post-transcriptional control of the signaling pathway and strengthened by the similar expression pattern of *IML1*, *NPR1*, *NPR3* that are members of the SEACIT complex which inhibits TORC1 in response to amino acid deprivation [25]. The enrichment in genes involved in respiration and mitochondrial functions (*COX1*, *COB*, *COX2*, *SDH4*, *SDH3*, *SDH1*) was unexpected since yeast cells were fermenting in anaerobic conditions and not able to respire. Actually, this differential expression may result from a down-regulation of Tor signaling in nitrogen-limited fermentations since it has been shown that reduced Tor signaling upregulated mitochondrial genes expression [26]. It is notable that there is no enrichment in genes involved in stress response in these two clusters although some stress responsive genes (*GLG1* and *GAC1*) involved in glycogen biosynthesis are found in cluster 24. Besides, *YGK3* that encodes a protein homologous to Mck1p (a key controller of both the entry in quiescent state and cell viability [24]) was found in cluster 10 and therefore only overexpressed in those two conditions that preserve viability. Other stress genes, not found in any clusters, were however also differentially expressed in the two sets of conditions as for example *WHI2* which has been reported to play a role both in stress response and cell death (S8 Fig). To obtain more information on stress (or stress-related) gene expression, we looked into some of them displayed in Fig 7. Most of the stress genes are equally induced in conditions leading to cell death although with some variations in their expression patterns. Some genes have a delayed or weaker expression in some conditions but the profile is not common to all micronutrient-limited fermentations. We observed that the key gene for protection against ROS, the mitochondrial superoxide dismutase *SOD2* [27], displayed a lower or delayed expression in conditions associated to cell death (Fig 7F). Actually, even if alcoholic fermentation happens under anaerobiosis, it has been proposed that the oxidative stress could play a role in alcoholic fermentation [28,29]. More precisely, this overexpression of *SOD2* could be more related to the ethanol stress-induced programmed cell death which has been shown to be mediated by mitochondria and by a causal role of ROS [29,30]. *MSN2* and *MSN4*, the two main stress transcriptional factors, are overexpressed under all the conditions (Fig 7G & 7H). The only exception is for nicotinic acid starvation under which *MSN4* expression was clearly repressed but seems to be counteracted by *MSN2* overexpression of in this same condition.

Only small clusters were found with genes displaying a reverse behaviour, that is to say a low expression for the two conditions supporting high viability and an induction in the conditions leading to yeast cell death. Cluster 14 (Fig 6C & S9 Fig for details) contains some genes displaying such an opposite pattern and is enriched in sulfur metabolism (*MET3*,*10*,*16* and *CYS4*) and amino acids biosynthesis genes (*LYS14*,*20*,*21*). The availability of nitrogen sources in the fermentation limited by micronutrients can explain part of the response and the expression of amino acid biosynthetic pathway.

This cluster contains also some genes more specific to nicotinic acid and pantothenic acid starvations. It includes genes involved in the biosynthesis and transport of NAD (*BNA1*, *BNA4*, *NRT1*) and overexpressed under such conditions. Their strong expression is consistent with nicotinic acid limitation and suggests that pantothenic acid-limited cells experience a similar metabolic state. Among the genes with a similar pattern, *RNR4* encoding a ribonucleotide reductase induced by DNA damage could point out to a DNA replication stress since it has been shown to be associated with cell death [31].

## Discussion

Understanding the mechanisms underlying yeast cell death during wine alcoholic fermentation is of key importance to improve fermentation management and prevent sluggish or stuck fermentations. Yeast cell death is known to occur under lipid limitation conditions combined to strong anaerobiosis and is usually considered only as a consequence of membrane dysfunction due to lipids defects. In a previous work [6], it was shown that ergosterol limitations in musts were leading to yeast cell death in a nitrogen-controlled manner, pointing out the key role of nitrogen signaling in such cell death. Based on this observation we have examined here whether other nutrients limitations could trigger yeast cell death in alcoholic fermentation in a similar manner. We focused our analyses on micronutrients such as vitamins and growth factors since they were more likely to correspond to unusual nutrient limitations for yeast, by contrast to phosphate, carbohydrate or sulfate, starvations which yeast has adapted to through evolution [4]. Among the 7 micronutrients checked in this study, we show that 4 trigger yeast cell death when they are in growth-restricting amounts in a nitrogen-rich medium. Two of them (ergosterol, oleic acid) are lipid growth factors conditional to anaerobic growth and the other two are vitamins, one (pantothenic acid) being involved in lipid metabolism. Cell death was therefore observed with some of the micronutrients for which yeast is auxotroph but not with others (thiamine, biotin) indicating that auxotrophy is not systematically associated with cell death in these conditions. Yeast obviously adapted to starvation for these latter compounds. Conversely, ergosterol, oleic acid, pantothenic and nicotinic acid clearly correspond to unusual nutrients limitations for yeast.

The results obtained here show that yeast cell death triggered by all these four micronutrients limitations depends on nitrogen availability. In all cases, excess nitrogen prevented the triggering of a starvation mechanism that maintains yeast cells alive. Conversely, lowering nitrogen level restored yeast cell viability. Our experimental set-up, by keeping a constant cell population, ruled out the possibility that nitrogen could act through a modulation of yeast biomass and available, per-cell quantity of micronutrient. Cell death occurrence was only dependent on the conditions of entry into starvation. These observations are consistent with the idea that an arrest of growth induced by nitrogen limitation leads to a state of resistance to starvation. It is also in line with the report of Boer et al [4] showing that the first limiting nutrient triggering the entry into stationary phase determines the fate and survival ability of yeast cells.

Given the impact of nitrogen on cell death, we explored the contribution of the nitrogen signaling pathway on yeast response. We observed that decreasing TORC1/*SCH9* signaling pathways restored high cell viability in several micronutrient-limited fermentations: viability was restored by *SCH9* deletion in oleic acid, ergosterol and pantothenic acid starvations while rapamycin prevented cell death in the first two situations. The inconsistency between the response to *SCH9* deletion and rapamycin addition under pantothenic acid limitation was unexpected and the toxic effect observed with the drug suggests that some other phenomenon may have interfered in these specific and physiologically extreme conditions. The inefficiency of *SCH9* deletion and rapamycin addition in nicotinic acid limitation is also puzzling and suggests that TORC1/*SCH9* signaling was somehow bypassed and that other signaling actors were involved. Nicotinic acid is connected to NAD metabolism which is itself involved in the regulation of cell longevity [32]. NAD and derived compounds have been shown to modulate various cellular function including sirtuin activity and yeast cells ageing [33]. Whether cross-regulation of these activities with nitrogen signaling can take place deserves additional investigations.

Although a deviation from the standard signaling scheme may have to be considered in this latter case, all the other situations are in line with an involvement of TORC1/*SCH9* signaling in cell death as previously described in various contexts [5,6,26,34,35]. Indeed this scheme is consistent with the transcriptomic analyses, which revealed a clear differential expression of a large set of genes that are targets of the TORC1/*SCH9* signaling pathway (*OPT1*, *OPT2*, *MEP2* or *NPR2*) in conditions leading to cell death compared to those leading to survival. A high signaling pathway activity likely prevents the adaptation of yeast cells to starvation in micronutrient-limited fermentations.

A key aspect of the adaptation to starvation is thought to be the triggering of an appropriate stress response. Our data show that the diverse nutrient starvations did not trigger an identical stress response. Yeast cells under nitrogen-limited conditions display a stress response as shown by their heat shock resistance and by their accumulation of glycogen. Indeed, stress resistance and storage of glycogen are features of quiescent cells and consistent with their ability to survive starvation. This ability to survive throughout fermentation was well correlated with stress resistance but not with the cell cycle status that exhibited heterogeneous perturbations in situations associated with cell death. By contrast, yeast cells in fermentations limited for oleic acid, ergosterol, pantothenic acid and nicotinic acid did not display such stress resistance. We thus attempted to determine whether differences in stress gene expression could be observed in transcriptomic data. Surprisingly, we did not find specific enrichments in stress genes in any dataset whatever the type of starvation. Specifically, stress-responsive genes were not detected, except for some isolated ones, in those clusters that differentiated the conditions allowing maintenance of viability from those leading to cell death. We therefore examined individually the expression patterns of a large set of stress genes. All these genes are involved in the overall stress response under the control of *MSN2/MSN4* indicating that a global stress response took place in all conditions whatever was the yeast growth-limiting nutrient limiting.

Therefore the activity of the nitrogen-signaling pathway did not block the expression of stress genes. This apparent stress response in micronutrients limited conditions is not consistent with the lack of resistance of yeast cells. This discrepancy suggests that the stress response is restricted at a post-transcriptional level in the micronutrients limited fermentations. The involvement of posttranscriptional control in the entry into quiescence and the acquisition of stress resistance following nutrient limitation have been described [36]. Such regulations could explain the lack of stress resistance despite the expression of stress genes. This is consistent with the observation that *SCH9* deletion triggered an increased yeast viability of ergosterol-limited cells but only triggered an overexpression of some selected stress genes [6].

Alternatively, some stress genes regulations critical for the adaptation to starvation might be independent of the general stress response pathway and be altered in micronutrients-limited fermentations. Such an independent control of stress gene expression was proposed for the regulator gene *WHI2* that has been shown to link nutrient sensing to cell death [37,38]. Interestingly *WHI2* displayed a clear differential expression between conditions leading to survival or cell death with respectively high and low expression level. A further characterization (in conditions similar to ours) of its putative role in cell death control could be therefore relevant. During the stationary phase of alcoholic fermentation, yeast cells experience a chronological ageing under conditions that combine multiple stress, the major one being ethanol. Other metabolites formed by yeast, i.e. acetaldehyde and acetic acid, have also been shown to impact cell ageing [39]. Indeed, these compounds produced during alcoholic fermentation are known to induce apoptosis-like cell death as it has been shown for acetic acid [40]. More importantly, ethanol is known to induce its own specific programmed cell death, closely related to apoptosis but mediated by the mitochondrial pathway [29,30]. The overexpression of some mitochondrial genes in our condition could be consistent with an ethanol-induced programmed cell death. In order to avoid such a premature ageing of cells in starvation, yeast cells require the triggering of an appropriate stress response.

We have shown here that several micronutrient limitations do not allow for such adaptation and are associated with cell death. It is likely that these nutrient limitations are not frequently encountered by yeast in natural environments although they can occur in industrial winemaking. Limitations for both ergosterol and oleic acid are rather common in grape musts following clarification processes prior to fermentation (in white or rosé wine fermentation). Variations in pantothenic acid content in grape musts have been reported by Hagen [41] suggesting that limitation in pantothenic acid could also occur. It is worth noting that Wang [42] reported that yeast cells were more sensitive to pantothenic acid deficiency and displayed cell death when the nitrogen level was high, thus leading to stuck fermentations, while low levels of nitrogen did not lead to such issue. This behaviour is consistent with our data and the role we ascribe here to nitrogen in triggering cell death under micronutrient limitation.

Our results provide a novel frame and should help optimizing nutrient management strategies in alcoholic fermentations. The addition of nitrogen, usually as NH_4_^+^, is a current practice in alcoholic fermentation that should benefit from the integration of possible interactions with other nutrients limitations. The results obtained were discussed here in relation to wine alcoholic fermentation only but it is likely that they can be applied to any other alcoholic fermentations situations (beer brewing, sake, etc.) and could help in the management of yeast cells death under many situations.

## Supporting information

S1 Table Time of fermentation in hour where the T1, T2, T3 and T4 samples were harvested for the different nutrient starvation conditions. Values of time are presented as mean of five sampling time ± standard deviation.
(PDF)

S1 Fig Viable cells (%) from *S*. *cerevisiae* Lalvin EC1118^®^ strain during alcoholic fermentation in a complete SM425 medium (high nitrogen) and in a SM425 medium limited in: (A) thiamin at 15 μg/L (high nitrogen / low thiamin); (B) biotin at 0.06 μg/L (high nitrogen / low biotin) and (C) inositol at 0.2 mg /L (high nitrogen / low inositol). (PDF)

S2 Fig Medium residual sugar during alcoholic fermentation by *S*. *cerevisiae* Lalvin EC1118^®^ under various micronutrient starvations. (A) (green curves) Ole-: Oleic acid starvation (18 mg/L), (B) (dark blue curves) Erg-: ergosterol starvation (1.5 mg/L), (C) (light blue curves) Pan-: pantothenic acid starvation (0.02 mg/L) and (D) (yellow curves) Nic-: nicotinic acid starvation (0.08 mg/L). Open circles indicate N-: low nitrogen (71 mg/L YAN); full diamonds indicate N+: high nitrogen (425 mg/L YAN). Residual sugar amount was calculated from released CO_2_ using linear regression (1 g of sugar consumed = 0.47 g of CO_2;_ [43]. Fermentations were performed in duplicate, error bars correspond to the standard deviation.
(PDF)

S3 Fig Evolution of the cell population as a function of alcoholic fermentation duration. For high or low nitrogen level under: (A) (green curves) Ole-: Oleic acid starvation (18 mg/L), (B) (dark blue curves) Erg-: ergosterol starvation (1.5 mg/L), (C) (light blue curves) Pan-: pantothenic acid starvation (0.02 mg/L) and (D) (yellow curves) Nic-: nicotinic acid starvation (0.08 mg/L). Open circles indicate N-: low nitrogen (71 mg/L YAN); full diamonds indicate N+: high nitrogen (425 mg/L YAN). Fermentations were performed in duplicate.
(PDF)

S4 Fig Comparison of the amount of residual assimilable nitrogen (YAN) at different time points in the fermentation medium. For the 6 nutrient conditions set up for the transcriptomic analysis. Measurements were performed at different times during alcoholic fermentation (T1, 20 10^6^ cells/mL; T2, 12 g CO_2_ produced; T3, 40 g CO_2_ produced; T4, 75 g CO_2_ produced) for: N-: low nitrogen, 71 mg/L YAN; N-/Erg-: low nitrogen/low ergosterol, 71 mg/L YAN, 1.5 mg/L ergosterol; N+/Ole-: high nitrogen/ low oleic acid, 425 mg/L YAN, 18 mg/L oleic acid; N+/Erg-: high nitrogen/ low ergosterol, 425 mg/L YAN, 1.5 mg/L ergosterol; N+/Pan-: high nitrogen / low pantothenic acid, 425 mg/L YAN, 0.02 mg/L pantothenic acid and N+/Nic-: high nitrogen/ low nicotinic acid, 425 mg/L YAN, 0.08 mg/L nicotinic acid. Yeast assimilable concentration was calculated from ammonium and free amino acid concentrations. Ammonium concentration was determined enzymatically (RBiopharm AG^™^, Darmstadt, Germany). The free amino acid content in the must was determined by cation exchange chromatography, with post-column ninhydrin derivatization (Biochrom 30, Biochrom^™^, Cambridge, UK) as previously described [44]. Fermentations were performed in duplicate, error bars are corresponding to the standard deviation.
(PDF)

S5 Fig Pattern of (A) cell viability (%), (B) cell population and (C) fermentation rates during alcoholic fermentation using the Lalvin EC1118^®^ strain. For the six nutrient conditions set up for transcriptome analysis: (red curves) N-: low nitrogen (71 mg/L YAN); (pink curves) N-/Erg-: low nitrogen/ low ergosterol (71 mg/L YAN, 1.5 mg/L ergosterol); (green curves) N+/Ole-: high nitrogen/ low oleic acid (425 mg/L YAN, 18 mg/L oleic acid); (dark blue curves) N+/Erg-: high nitrogen/ low ergosterol (425 mg/L YAN, 1.5 mg/L ergosterol); (light blue curves) N+/Pan-: high nitrogen / low pantothenic acid (425 mg/L YAN, 0.02 mg/L pantothenic acid); and (yellow curves) N+/Nic-: high nitrogen/ low nicotinic acid (425 mg/L YAN, 0.08 mg/L nicotinic acid). Curves show the mean of fermentations performed in duplicate for cell viability and triplicate for cell population and fermentation rate. Error bars correspond to the standard deviation.
(PDF)

S6 Fig Genes with low expression in micronutrient starvation compared with nitrogen starvation (cluster 10) during alcoholic fermentation. For: N-: low nitrogen, 71 mg/L YAN; N-/Erg-: low nitrogen/low ergosterol, 71 mg/L YAN, 1.5 mg/L ergosterol; N+/Ole-: high nitrogen/ low oleic acid, 425 mg/L YAN, 18 mg/L oleic acid; N+/Erg-: high nitrogen/ low ergosterol, 425 mg/L YAN, 1.5 mg/L ergosterol; N+/Pan-: high nitrogen/ low pantothenic acid, 425 mg/L YAN, 0.02 mg/L pantothenic acid and N+/Nic-: high nitrogen/ low nicotinic acid, 425 mg/L YAN, 0.08 mg/L nicotinic acid; transcriptomic assays were performed at four time points during alcoholic fermentation (T1, 20 10^6^ cells/mL; T2, 12 g CO_2_ produced; T3, 40 g CO_2_ produced; T4, 75 g CO_2_ produced) indicated by the grey triangle. Results show the mean of biological triplicates.
(PDF)

S7 Fig Genes with low expression in micronutrient starvations compare with nitrogen starvation (cluster 24) during alcoholic fermentation. For: N-: low nitrogen, 71 mg/L YAN; N-/Erg-: low nitrogen/low ergosterol, 71 mg/L YAN, 1.5 mg/L ergosterol; N+/Ole-: high nitrogen/ low oleic acid, 425 mg/L YAN, 18 mg/L oleic acid; N+/Erg-: high nitrogen/ low ergosterol, 425 mg/L YAN, 1.5 mg/L ergosterol; N+/Pan-: high nitrogen/ low pantothenic acid, 425 mg/L YAN, 0.02 mg/L pantothenic acid and N+/Nic-: high nitrogen/ low nicotinic acid, 425 mg/L YAN, 0.08 mg/L nicotinic acid; transcriptomic assays were performed at four time points during alcoholic fermentation (T1, 20 10^6^ cells/mL; T2, 12 g CO_2_ produced; T3, 40 g CO_2_ produced; T4, 75 g CO_2_ produced) indicated by the grey triangle. Results show the mean of biological triplicates.
(PDF)

S8 Fig Kinetics of the expression (arbitrary units) of the *WHI2* stress gene during alcoholic fermentation under 6 nutrient starvation conditions. (red curves) N-: low nitrogen (71 mg/L YAN); (pink curves) N-/Erg-: low nitrogen/ low ergosterol (71 mg/L YAN, 1.5 mg/L ergosterol); (green curves) N+/Ole-: high nitrogen/ low oleic acid (425 mg/L YAN, 18 mg/L oleic acid); (dark blue curves) N+/Erg-: high nitrogen/ low ergosterol (425 mg/L YAN, 1.5 mg/L ergosterol); (light blue curves) N+/Pan-: high nitrogen / low pantothenic acid (425 mg/L YAN, 0.02 mg/L pantothenic acid); and (yellow curves) N+/Nic-: high nitrogen/ low nicotinic acid (425 mg/L YAN, 0.08 mg/L nicotinic acid). Curves show the mean of biological triplicates, error bars correspond to the standard deviation.
(PDF)

S9 Fig Highly expressed genes during micronutrient starvations compared with nitrogen starvations and genes specific to pantothenic acid and nicotinic acid starvation (cluster 14) during alcoholic fermentation. For: N-: low nitrogen, 71 mg/L YAN; N-/Erg-: low nitrogen/low ergosterol, 71 mg/L YAN, 1.5 mg/L ergosterol; N+/Ole-: high nitrogen/ low oleic acid, 425 mg/L YAN, 18 mg/L oleic acid; N+/Erg-: high nitrogen/ low ergosterol, 425 mg/L YAN, 1.5 mg/L ergosterol; N+/Pan-: high nitrogen/ low pantothenic acid, 425 mg/L YAN, 0.02 mg/L pantothenic acid and N+/Nic-: high nitrogen/ low nicotinic acid, 425 mg/L YAN, 0.08 mg/L nicotinic acid; transcriptomic assays were performed at four time points during alcoholic fermentation (T1, 20 10^6^ cells/mL; T2, 12 g CO_2_ produced; T3, 40 g CO_2_ produced; T4, 75 g CO_2_ produced) indicated by the grey triangle. Results show the mean of biological triplicates.
(PDF)
