# Supplementary material for: A set of nutrient limitations trigger yeast cell death in a nitrogen-dependent manner during wine alcoholic fermentation
Source: PLoS One. 2017 Sep 18;12(9):e0184838. doi: 10.1371/journal.pone.0184838 (PMC5602661; doi:10.1371/journal.pone.0184838)
Supplement: S1 Table — Values of time are presented as mean of five sampling time ± standard deviation. (PDF) [file pone.0184838.s001.pdf]

S1 Table. Time of fermentation in hour where the T1, T2, T3 and T4 samples were harvested under the different nutrient starvation conditions. Values of time are presented as mean of five sampling time ± standard deviation.

	**T1** (20 10^6 cells/mL)	**T2** (12 g of CO_2 produced)	**T3** (40 g of CO_2 produced)	**T4** (75 g of CO_2 produced)
**N-**	19.27 ± 0.2	47.64 ± 1.84	138.29 ± 9.36	281.71 ± 17.82
**N-/Erg-**	19.26 ± 0.2	47.97 ± 0.27	145.08 ± 1.49	291.32 ± 1.07
**N+/Ole-**	19.58 ± 0.52	30.80 ± 0.36	54.27 ± 1.23	105.58 ± 3.76
**N+/Erg-**	19.15 ± 0.37	30.39 ± 0.94	55.39 ± 2.27	97.98 ± 4.44
**N+/Pan-**	19.40 ± 0.36	34.62 ± 1.17	83.33 ± 3.81	300.84 ± 60.75
**N+/Nic-**	18.59 ± 0.20	35.60 ± 1.52	104.44 ± 4.23	399.31 ± 12.03
