# Supplementary material for: A set of nutrient limitations trigger yeast cell death in a nitrogen-dependent manner during wine alcoholic fermentation
Source: PLoS One. 2017 Sep 18;12(9):e0184838. doi: 10.1371/journal.pone.0184838 (PMC5602661; doi:10.1371/journal.pone.0184838)
Supplement: S1 Fig — (PDF) [file pone.0184838.s002.pdf]

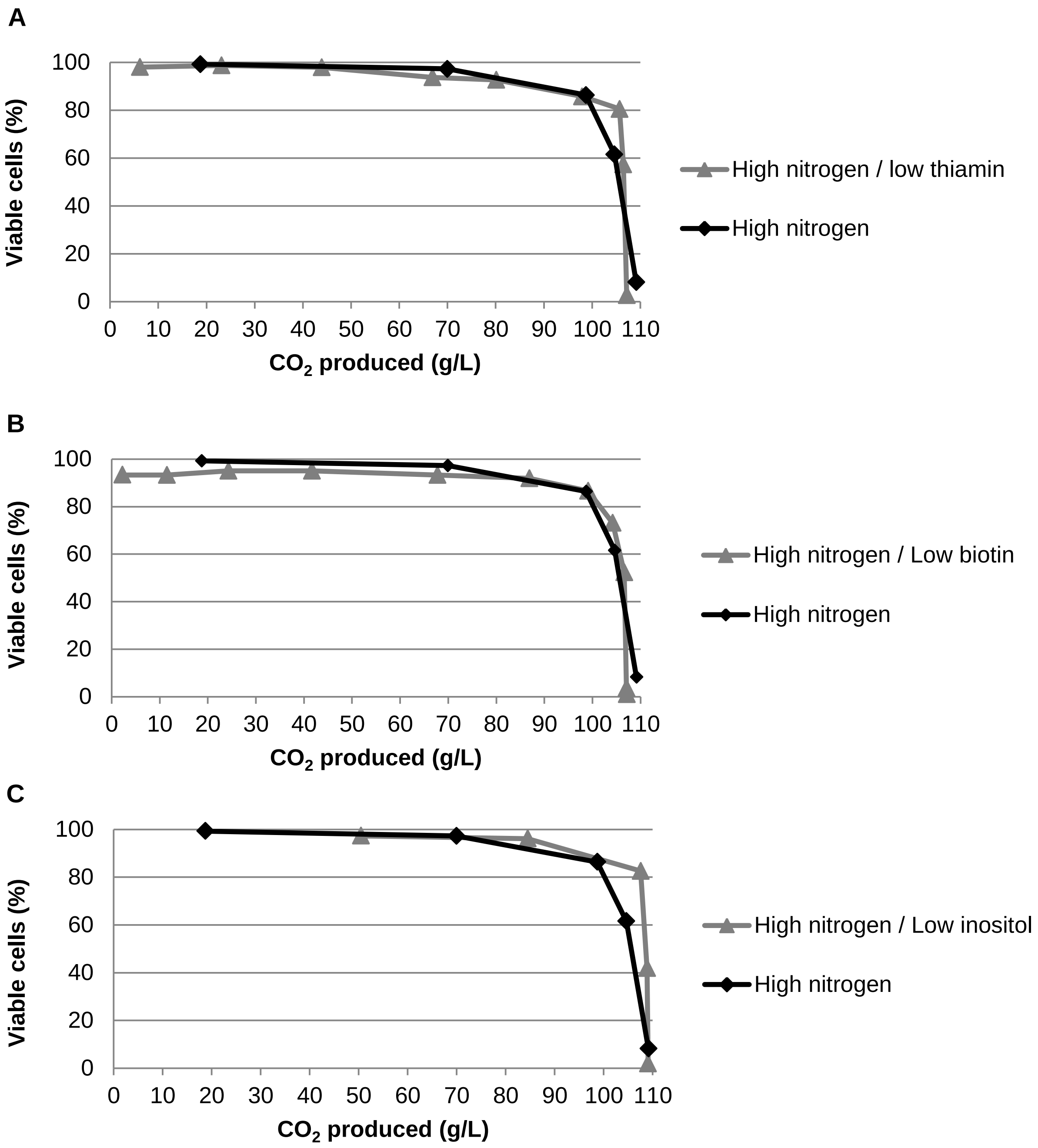

S1 Fig. Viable cells (%) from *S.cerevisiae* Lalvin EC1118® strain during alcoholic fermentation in a complete SM425 medium (High nitrogen) and in a SM425 medium limited in: (A) thiamin at 15 µg/L (High nitrogen / low thiamin); (B) biotin at 0.06 µg/L (High nitrogen / Low biotin) and (C) inositol at 0.2 mg /L (High nitrogen / Low inositol).
