# Supplementary material for: A set of nutrient limitations trigger yeast cell death in a nitrogen-dependent manner during wine alcoholic fermentation
Source: PLoS One. 2017 Sep 18;12(9):e0184838. doi: 10.1371/journal.pone.0184838 (PMC5602661; doi:10.1371/journal.pone.0184838)
Supplement: S3 Fig — For high or low nitrogen level under: (A) (green curves) Ole-: Oleic acid starvation (18 mg/L), (B) (dark blue curves) Erg-: ergosterol starvation (1.5 mg/L), (C) (light blue curves) Pan-: pantothenic acid starvation (0.02 mg/L) and (D) (yellow curves) Nic-: nicotinic acid starvation (0.08 mg/L). Open circles indicate N-: low nitrogen (71 mg/L YAN); full diamonds indicate N+: high nitrogen (425 mg/L YAN). Fermentations were performed in duplicate. (PDF) [file pone.0184838.s004.pdf]

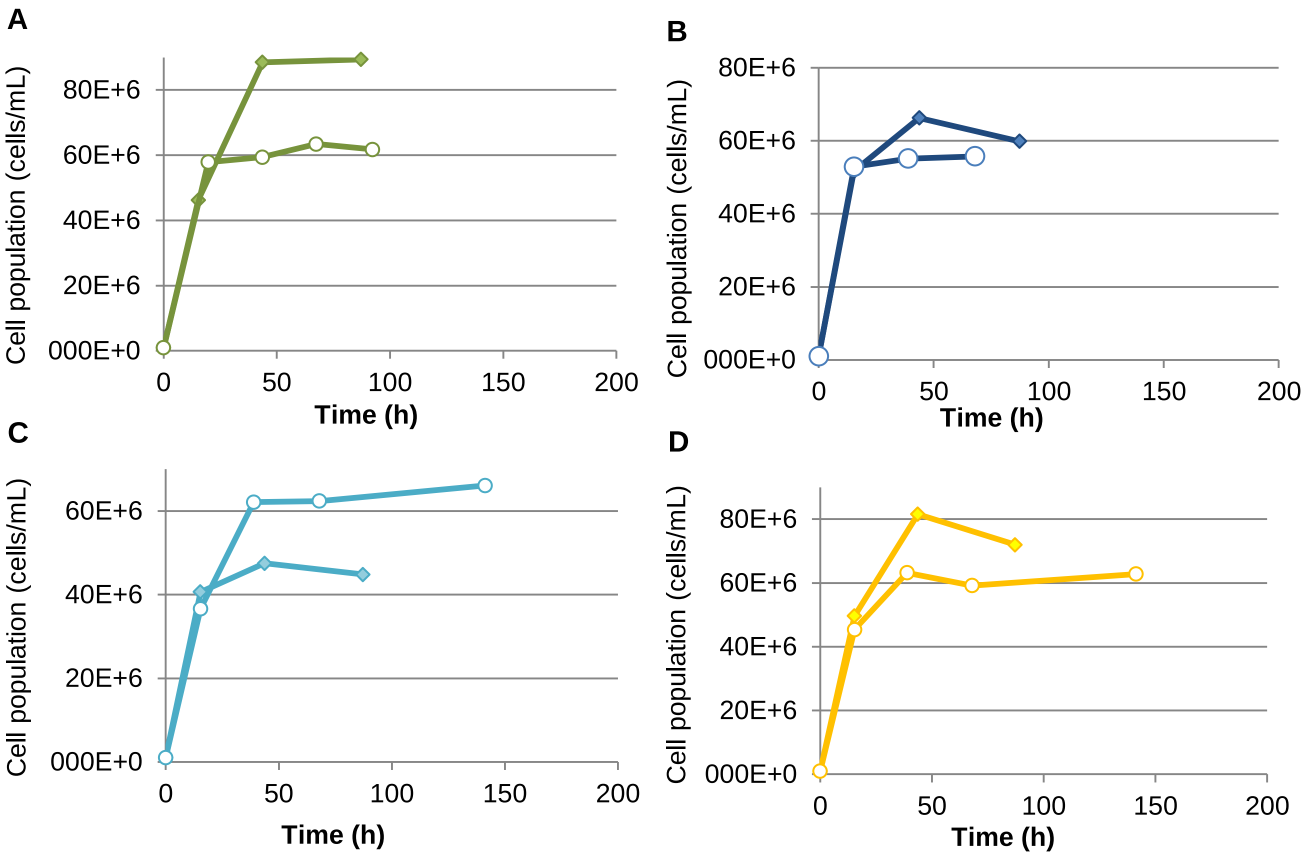

S3 Fig. Evolution of the cell population as a function of alcoholic fermentation duration at high or low nitrogen level under : (A) (▬) Ole-: Oleic acid starvation (18 mg/L), (B) (▬) Erg-: ergosterol starvation (1.5 mg/L), (C) (▬) Pan-: pantothenic acid starvation (0.02 mg/L) and (D) (▬) Nic-: nicotinic acid starvation (0.08 mg/L). (○,○,○,○) indicate N-: low nitrogen (71 mg/L YAN); (♦,♦,♦,♦) indicate N+: high nitrogen (425 mg/L YAN). Fermentations were performed in duplicate.
