# Supplementary material for: A set of nutrient limitations trigger yeast cell death in a nitrogen-dependent manner during wine alcoholic fermentation
Source: PLoS One. 2017 Sep 18;12(9):e0184838. doi: 10.1371/journal.pone.0184838 (PMC5602661; doi:10.1371/journal.pone.0184838)
Supplement: S4 Fig — For the 6 nutrient conditions set up for the transcriptomic analysis. Measurements were performed at different times during alcoholic fermentation (T1, 20 106 cells/mL; T2, 12 g CO2 produced; T3, 40 g CO2 produced; T4, 75 g CO2 produced) for: N-: low nitrogen, 71 mg/L YAN; N-/Erg-: low nitrogen/low ergosterol, 71 mg/L YAN, 1.5 mg/L ergosterol; N+/Ole-: high nitrogen/ low oleic acid, 425 mg/L YAN, 18 mg/L oleic acid; N+/Erg-: high nitrogen/ low ergosterol, 425 mg/L YAN, 1.5 mg/L ergosterol; N+/Pan-: high nitrogen / low pantothenic acid, 425 mg/L YAN, 0.02 mg/L pantothenic acid and N+/Nic-: high nitrogen/ low nicotinic acid, 425 mg/L YAN, 0.08 mg/L nicotinic acid. Yeast assimilable concentration was calculated from ammonium and free amino acid concentrations. Ammonium concentration was determined enzymatically (RBiopharm AG™, Darmstadt, Germany). The free amino acid content in the must was determined by cation exchange chromatography, with post-column ninhydrin derivatization (Biochrom 30, Biochrom™, Cambridge, UK) as previously described [44]. Fermentations were performed in duplicate, error bars are corresponding to the standard deviation. (PDF) [file pone.0184838.s005.pdf]

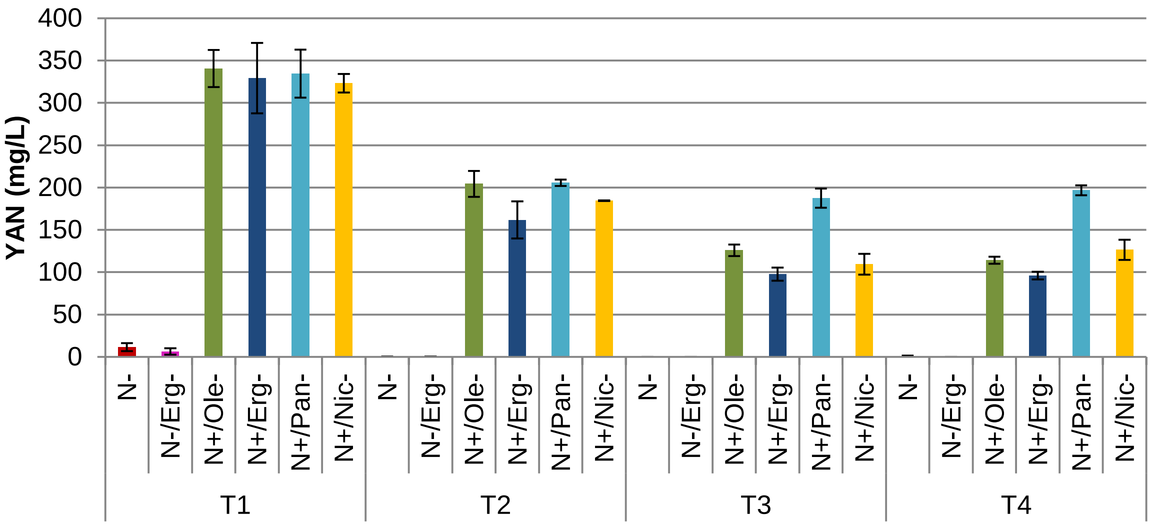

S4 Fig. Comparison of the amount of residual assimilable nitrogen (YAN) at different time points in the fermentation medium for the 6 nutrient conditions set up for the transcriptomic analysis

Measurements were performed at different times during alcoholic fermentation (T1, 20 10^6 cells/mL; T2, 12 g CO_2 produced; T3, 40 g CO_2 produced; T4, 75 g CO_2 produced) for : N- : low nitrogen, 71 mg/L YAN; N-/Erg- : low nitrogen/low ergosterol, 71 mg/L YAN, 1.5 mg/L ergosterol; N+/Ole-: high nitrogen/ low oleic acid, 425 mg/L YAN, 18 mg/L oleic acid; N+/Erg- : high nitrogen/ low ergosterol, 425 mg/L YAN, 1.5 mg/L ergosterol; N+/Pan- : high nitrogen / low pantothenic acid, 425 mg/L YAN, 0.02 mg/L pantothenic acid and N+/Nic- : high nitrogen/ low nicotinic acid, 425 mg/L YAN, 0.08 mg/L nicotinic acid. Yeast assimilable concentration was calculated from ammonium and free amino acid concentrations. Ammonium concentration was determined enzymatically (RBiopharm AG™, Darmstadt, Germany). The free amino acid content in the must was determined by cation exchange chromatography, with post-column ninhydrin derivatization (Biochrom 30, Biochrom™, Cambridge, UK) as previously described (41). Results are the mean of biological duplicate. Error bars correspond to standard deviation.
