# Supplementary material for: A set of nutrient limitations trigger yeast cell death in a nitrogen-dependent manner during wine alcoholic fermentation
Source: PLoS One. 2017 Sep 18;12(9):e0184838. doi: 10.1371/journal.pone.0184838 (PMC5602661; doi:10.1371/journal.pone.0184838)
Supplement: S5 Fig — For the six nutrient conditions set up for transcriptome analysis: (red curves) N-: low nitrogen (71 mg/L YAN); (pink curves) N-/Erg-: low nitrogen/ low ergosterol (71 mg/L YAN, 1.5 mg/L ergosterol); (green curves) N+/Ole-: high nitrogen/ low oleic acid (425 mg/L YAN, 18 mg/L oleic acid); (dark blue curves) N+/Erg-: high nitrogen/ low ergosterol (425 mg/L YAN, 1.5 mg/L ergosterol); (light blue curves) N+/Pan-: high nitrogen / low pantothenic acid (425 mg/L YAN, 0.02 mg/L pantothenic acid); and (yellow curves) N+/Nic-: high nitrogen/ low nicotinic acid (425 mg/L YAN, 0.08 mg/L nicotinic acid). Curves show the mean of fermentations performed in duplicate for cell viability and triplicate for cell population and fermentation rate. Error bars correspond to the standard deviation. (PDF) [file pone.0184838.s006.pdf]

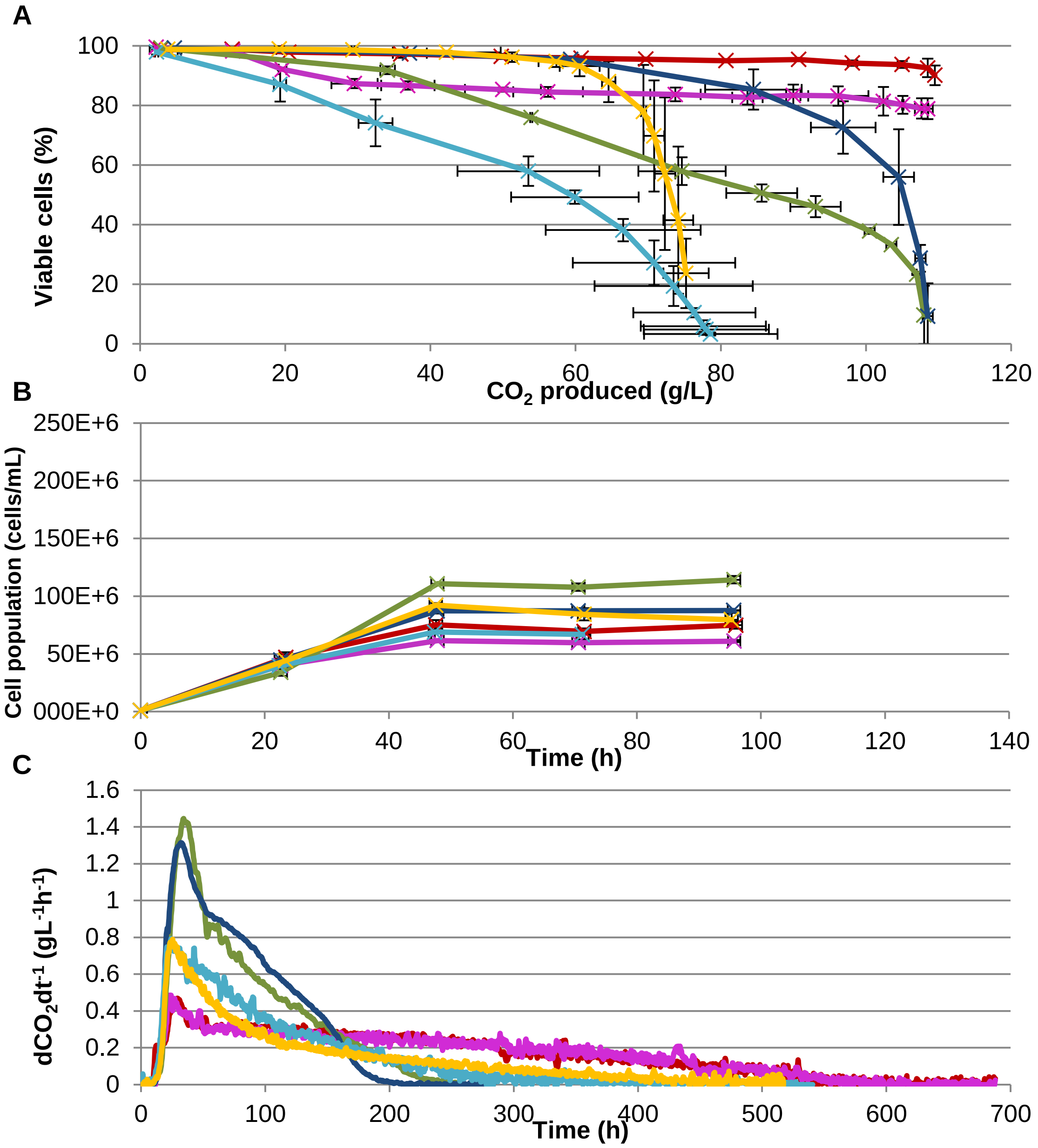

S5 Fig. Pattern of (A) cell viability, (B) cell population and (C) fermentation rates during alcoholic fermentation using the Lalvin EC1118® strain for the six nutrient conditions set up for transcriptome analysis

(—) N-: Low nitrogen (71 mg/L YAN); (—) N-/Erg-: Low nitrogen/ Low ergosterol (71 mg/L YAN, 1.5 mg/L ergosterol); (—) N+/Ole-: High nitrogen/ Low oleic acid (425 mg/L YAN, 18 mg/L oleic acid); (—) N+/Erg-: High nitrogen/ Low ergosterol (425 mg/L YAN, 1.5 mg/L ergosterol); (—) N+/Pan-: High nitrogen / Low pantothenic acid (425 mg/L YAN, 0.02 mg/L pantothenic acid); and (—) N+/Nic-: High nitrogen/ Low nicotinic acid (425 mg/L YAN, 0.08 mg/L nicotinic acid). Curves show the mean of fermentations performed in duplicate for cell viability and triplicate for cell population and fermentation rate. Error bars correspond to standard deviation.
