# Supplementary material for: A set of nutrient limitations trigger yeast cell death in a nitrogen-dependent manner during wine alcoholic fermentation
Source: PLoS One. 2017 Sep 18;12(9):e0184838. doi: 10.1371/journal.pone.0184838 (PMC5602661; doi:10.1371/journal.pone.0184838)
Supplement: S6 Fig — For: N-: low nitrogen, 71 mg/L YAN; N-/Erg-: low nitrogen/low ergosterol, 71 mg/L YAN, 1.5 mg/L ergosterol; N+/Ole-: high nitrogen/ low oleic acid, 425 mg/L YAN, 18 mg/L oleic acid; N+/Erg-: high nitrogen/ low ergosterol, 425 mg/L YAN, 1.5 mg/L ergosterol; N+/Pan-: high nitrogen/ low pantothenic acid, 425 mg/L YAN, 0.02 mg/L pantothenic acid and N+/Nic-: high nitrogen/ low nicotinic acid, 425 mg/L YAN, 0.08 mg/L nicotinic acid; transcriptomic assays were performed at four time points during alcoholic fermentation (T1, 20 106 cells/mL; T2, 12 g CO2 produced; T3, 40 g CO2 produced; T4, 75 g CO2 produced) indicated by the grey triangle. Results show the mean of biological triplicates. (PDF) [file pone.0184838.s007.pdf]

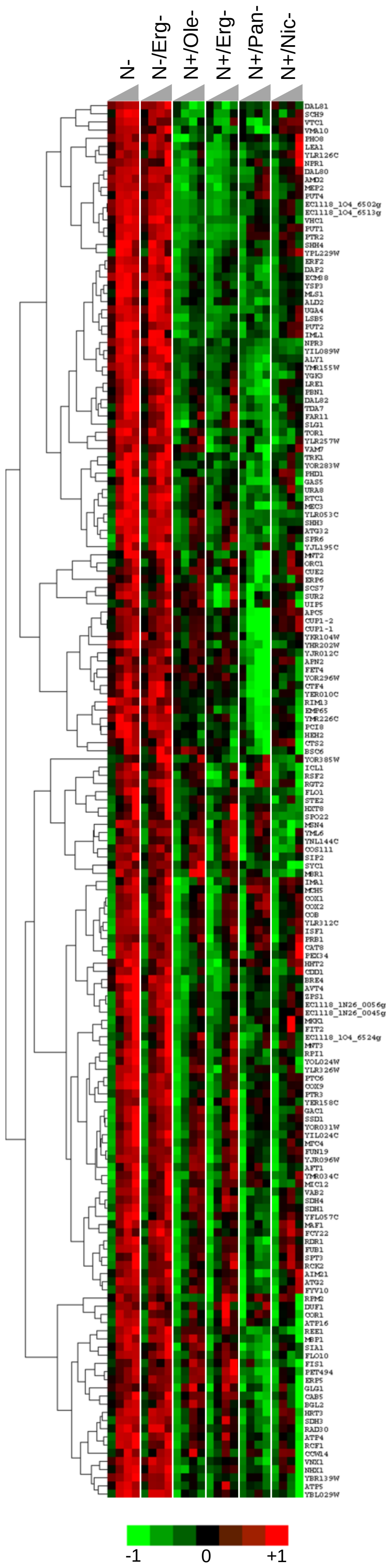

Category	p-value	In Category from Cluster	k	f
electron transport chain [GO:0022900]	2.713e-07	COX1 COB COX2 COR1 SDH4 SDH3 SDH1 YLR164W YMR118C SCS7	10	49
tricarboxylic acid cycle [GO:0006099]	6.992e-06	SDH4 ICL1 SDH3 SDH1 YLR164W YMR118C MLS1	7	30
proline metabolic process [GO:0006560]	6.128e-05	PUT2 PUT1 PUT4	3	4
mitochondrial electron transport, succinate to ubiquinone [GO:0006121]	0.0001504	SDH4 SDH3 SDH1	3	5
respiratory electron transport chain [GO:0022904]	0.0005071	COB COX2 COX9	3	7
detoxification of copper ion [GO:0010273]	0.0006283	CUP1-1 CUP1-2	2	2
proline catabolic process to glutamate [GO:0010133]	0.0006283	PUT2 PUT1	2	2
detoxification of cadmium ion [GO:0071585]	0.0006283	CUP1-1 CUP1-2	2	2
transport [GO:0006810]	0.0008894	COX1 COB COX2 COR1 YBR235W ATP16 RGT2 UGA4 BRE4 SDH4 ATP5 NHX1 FCY22 ERP6 VMA10 ERP5 TRK1 HXT8 SDH3 SDH1 PTR2 YKR104W YLR164W YMR118C SCS7 FET4 AVT4 MEP2 ATG2 VNX1 BSC6 MCH5 PUT4 FIT2 ATP4	35	815
cellular respiration [GO:0045333]	0.001172	SDH4 SDH3 SDH1	3	9
ion transport [GO:0006811]	0.001405	ATP16 ATP5 NHX1 VMA10 TRK1 FET4 VNX1 FIT2 ATP4	9	107
proline catabolic process [GO:0006562]	0.001854	PUT1 PUT4	2	3
gamma-aminobutyric acid transport [GO:0015812]	0.001854	UGA4 PUT4	2	3
proteolysis [GO:0006508]	0.002444	COR1 YBR139W PRB1 DAP2 RIM13 YGK3 YSP3	7	74
protein processing [GO:0016485]	0.002905	PBN1 DAP2 RIM13	3	12
mitochondrial electron transport, cytochrome c to oxygen [GO:0006123]	0.002905	COX1 COX2 COX9	3	12
proton transport [GO:0015992]	0.003407	ATP16 ATP5 VMA10 SIA1 ATP4	5	41
potassium ion transmembrane transport [GO:0071805]	0.003646	NHX1 TRK1	2	4
removal of superoxide radicals [GO:0019430]	0.003646	CUP1-1 CUP1-2	2	4
potassium ion transport [GO:0006813]	0.003707	NHX1 TRK1 VNX1	3	13
aerobic respiration [GO:0009060]	0.004083	COX1 COB COX2 COR1 MBR1 ISF1	6	61
age-dependent response to oxidative stress involved in chronological cell aging [GO:0001324]	0.005977	SCH9 MSN4	2	5
response to copper ion [GO:0046688]	0.005977	CUP1-1 CUP1-2	2	5
flocculation via cell wall protein-carbohydrate interaction [GO:0000501]	0.005977	FLO1 FLO10	2	5
flocculation [GO:0000128]	0.005977	FLO1 FLO10	2	5
ATP synthesis coupled proton transport [GO:0015986]	0.008187	ATP16 ATP5 ATP4	3	17
negative regulation of catalytic activity [GO:0043086]	0.008817	PRB1 YSP3	2	6
signal transduction [GO:0007165]	0.009784	COS111 RGT2 STE2 SIP2 TOR1 MKK1	6	73

S6 Fig. Genes with low expression in micronutrient starvation compared with nitrogen starvation (cluster 10) during alcoholic fermentation

For : N- **:** low nitrogen, 71 mg/L YAN; N-/Erg- : low nitrogen/low ergosterol, 71 mg/L YAN, 1.5 mg/L ergosterol; N+/Ole-: high nitrogen/ low oleic acid, 425 mg/L YAN, 18 mg/L oleic acid; N+/Erg-: high nitrogen/ low ergosterol, 425 mg/L YAN, 1.5 mg/L ergosterol; N+/Pan-: high nitrogen/ low pantothenic acid, 425 mg/L YAN, 0.02 mg/L pantothenic acid and N+/Nic-: high nitrogen/ low nicotinic acid, 425 mg/L YAN, 0.08 mg/L nicotinic acid; transcriptomic assays were performed at four time points during alcoholic fermentation (T1, 20 10^6 cells/mL; T2, 12 g CO_2 produced; T3, 40 g CO_2 produced; T4, 75 g CO_2 produced) indicated by (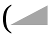). Results show the mean of biological triplicate.
