# Supplementary material for: A set of nutrient limitations trigger yeast cell death in a nitrogen-dependent manner during wine alcoholic fermentation
Source: PLoS One. 2017 Sep 18;12(9):e0184838. doi: 10.1371/journal.pone.0184838 (PMC5602661; doi:10.1371/journal.pone.0184838)
Supplement: S7 Fig — For: N-: low nitrogen, 71 mg/L YAN; N-/Erg-: low nitrogen/low ergosterol, 71 mg/L YAN, 1.5 mg/L ergosterol; N+/Ole-: high nitrogen/ low oleic acid, 425 mg/L YAN, 18 mg/L oleic acid; N+/Erg-: high nitrogen/ low ergosterol, 425 mg/L YAN, 1.5 mg/L ergosterol; N+/Pan-: high nitrogen/ low pantothenic acid, 425 mg/L YAN, 0.02 mg/L pantothenic acid and N+/Nic-: high nitrogen/ low nicotinic acid, 425 mg/L YAN, 0.08 mg/L nicotinic acid; transcriptomic assays were performed at four time points during alcoholic fermentation (T1, 20 106 cells/mL; T2, 12 g CO2 produced; T3, 40 g CO2 produced; T4, 75 g CO2 produced) indicated by the grey triangle. Results show the mean of biological triplicates. (PDF) [file pone.0184838.s008.pdf]

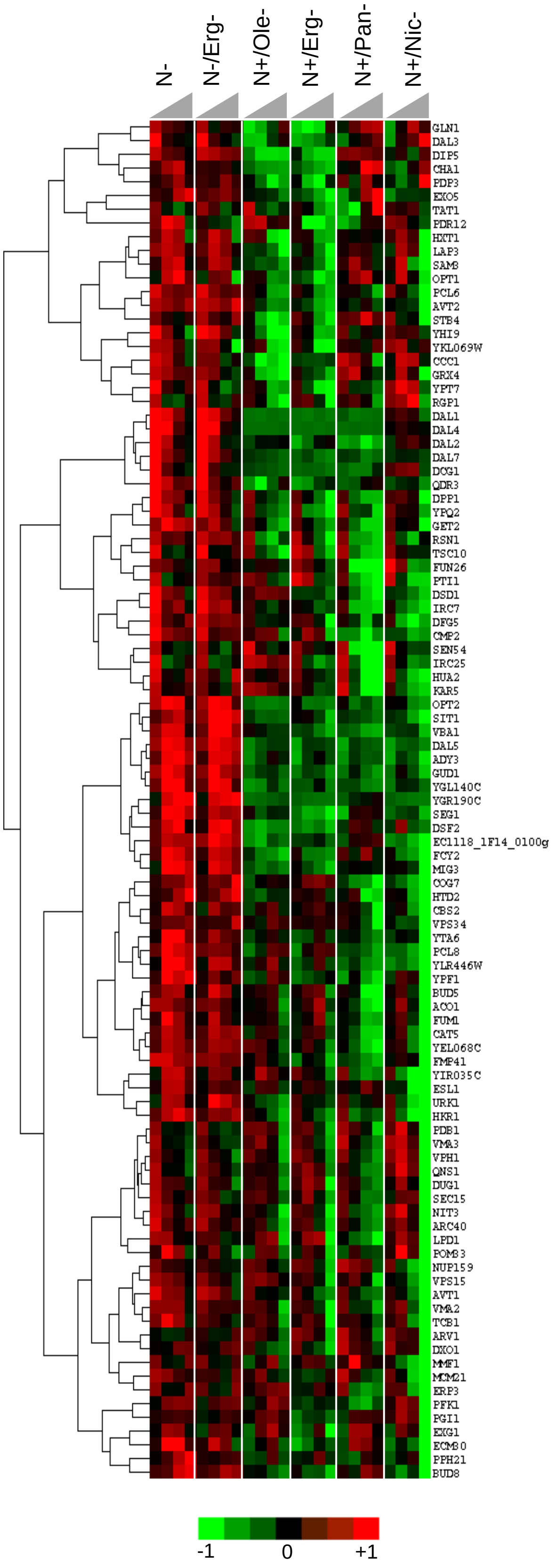

Category	p-value	In Category from Cluster	k	f
allantoin catabolic process [GO:0000256]	9.672e-09	DAL1 DAL4 DAL2 DAL7 DAL3	5	7
purine base metabolic process [GO:0006144]	2.036e-07	DAL1 DAL4 DAL2 DAL7 DAL3	5	11
transport [GO:0006810]	2.538e-07	FUN26 QDR3 TAT1 VPS15 VMA2 ERP3 VMA3 AVT2 SIT1 FCY2 GET2 COG7 SEC15 HXT1 NUP159 DAL4 OPT1 AVT1 DAL5 POM33 CCC1 VPS34 ARV1 YPT7 VBA1 VPH1 PDR12 DIP5 SAM3 OPT2	30	815
transmembrane transport [GO:0055085]	3.452e-06	FUN26 QDR3 TAT1 VMA2 SIT1 FCY2 HXT1 NUP159 DAL4 OPT1 DAL5 POM33 VBA1 DIP5 SAM3 OPT2	16	303
amino acid transport [GO:0006865]	2.175e-05	TAT1 AVT2 AVT1 VBA1 DIP5 SAM3	6	42
nitrogen compound metabolic process [GO:0006807]	3.176e-05	QNS1 DCG1 NIT3 GLN1	4	14
glycolysis [GO:0006096]	0.0005594	PGI1 PDB1 PFK1 YLR446W	4	28
cellular iron ion homeostasis [GO:0006879]	0.0008327	VMA3 SIT1 GRX4 CCC1	4	31
ATP hydrolysis coupled proton transport [GO:0015991]	0.001545	VMA2 VMA3 VPH1	3	17
peptide transport [GO:0015833]	0.001869	OPT1 OPT2	2	5
propionate metabolic process [GO:0019541]	0.001869	ACO1 PDR12	2	5
oligopeptide transport [GO:0006857]	0.001869	OPT1 OPT2	2	5
sulfur compound metabolic process [GO:0006790]	0.003853	IRC7 OPT1	2	7
amino acid transmembrane transport [GO:0003333]	0.004286	TAT1 DIP5 SAM3	3	24
cellular bud site selection [GO:0000282]	0.005397	BUD5 HKR1 BUD8	3	26
vacuolar acidification [GO:0007035]	0.005397	VMA2 VMA3 VPH1	3	26
cellular calcium ion homeostasis [GO:0006874]	0.006487	VMA2 CCC1	2	9
nucleobase transport [GO:0015851]	0.008036	FCY2 DAL4	2	10
tricarboxylic acid cycle [GO:0006099]	0.008096	DAL7 ACO1 FUM1	3	30
nucleobase, nucleoside, nucleotide and nucleic acid transport [GO:0015931]	0.009733	FCY2 DAL4	2	11
carbohydrate metabolic process [GO:0005975]	0.009759	PCL6 EXG1 YLR446W CAT5 PCL8	5	94

S7 Fig. Genes with low expression in micronutrient starvations compare with nitrogen starvation (cluster 24) during alcoholic fermentation

For : N- **:** low nitrogen, 71 mg/L YAN; N-/Erg- : low nitrogen/low ergosterol, 71 mg/L YAN, 1.5 mg/L ergosterol; N+/Ole-: high nitrogen/ low oleic acid, 425 mg/L YAN, 18 mg/L oleic acid; N+/Erg-: high nitrogen/ low ergosterol, 425 mg/L YAN, 1.5 mg/L ergosterol; N+/Pan-: high nitrogen/ low pantothenic acid, 425 mg/L YAN, 0.02 mg/L pantothenic acid and N+/Nic-: high nitrogen/ low nicotinic acid, 425 mg/L YAN, 0.08 mg/L nicotinic acid; transcriptomic assays were performed at four time points during alcoholic fermentation (T1, 20 10^6 cells/mL; T2, 12 g CO_2 produced; T3, 40 g CO_2 produced; T4, 75 g CO_2 produced) indicated by (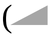). Results show the mean of biological triplicate.
