# Supplementary material for: A set of nutrient limitations trigger yeast cell death in a nitrogen-dependent manner during wine alcoholic fermentation
Source: PLoS One. 2017 Sep 18;12(9):e0184838. doi: 10.1371/journal.pone.0184838 (PMC5602661; doi:10.1371/journal.pone.0184838)
Supplement: S9 Fig — For: N-: low nitrogen, 71 mg/L YAN; N-/Erg-: low nitrogen/low ergosterol, 71 mg/L YAN, 1.5 mg/L ergosterol; N+/Ole-: high nitrogen/ low oleic acid, 425 mg/L YAN, 18 mg/L oleic acid; N+/Erg-: high nitrogen/ low ergosterol, 425 mg/L YAN, 1.5 mg/L ergosterol; N+/Pan-: high nitrogen/ low pantothenic acid, 425 mg/L YAN, 0.02 mg/L pantothenic acid and N+/Nic-: high nitrogen/ low nicotinic acid, 425 mg/L YAN, 0.08 mg/L nicotinic acid; transcriptomic assays were performed at four time points during alcoholic fermentation (T1, 20 106 cells/mL; T2, 12 g CO2 produced; T3, 40 g CO2 produced; T4, 75 g CO2 produced) indicated by the grey triangle. Results show the mean of biological triplicates. (PDF) [file pone.0184838.s010.pdf]

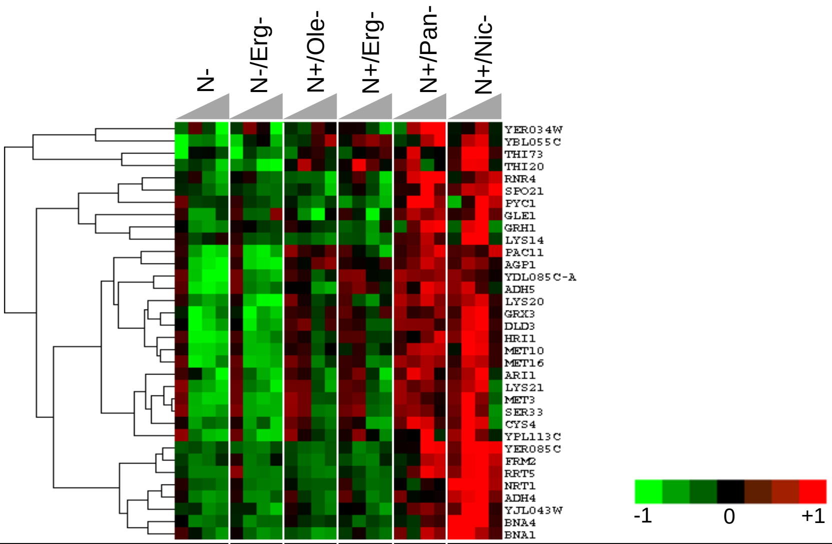

Category	p-value	In Category from Cluster	k	f
oxidation-reduction process [GO:0055114]	4.575e-09	BNA4 ADH5 FRM2 DLD3 MET10 ARI1 ADH4 RNR4 SER33 BNA1 YPL113C MET16	12	272
cellular amino acid biosynthetic process [GO:0008652]	2.331e-08	LYS21 LYS20 LYS14 MET10 CYS4 SER33 MET3 MET16	8	98
cysteine biosynthetic process [GO:0019344]	2.818e-07	MET10 CYS4 MET3 MET16	4	12
metabolic process [GO:0008152]	6.477e-07	BNA4 ADH5 LYS21 LYS20 MET10 PYC1 ARI1 CYS4 SER33 BNA1 YPL113C MET16	12	425
lysine biosynthetic process [GO:0009085]	6.865e-06	LYS21 LYS20 LYS14	3	8
lysine biosynthetic process via aminoadipic acid [GO:0019878]	6.865e-06	LYS21 LYS20 LYS14	3	8
sulfate assimilation [GO:0000103]	2.001e-05	MET10 MET3 MET16	3	11
de novo NAD biosynthetic process from tryptophan [GO:0034354]	0.0002549	BNA4 BNA1	2	5
amino acid catabolic process to alcohol via Ehrlich pathway [GO:0000947]	0.0003811	ADH5 ADH4	2	6
methionine biosynthetic process [GO:0009086]	0.0005082	MET10 MET3 MET16	3	31
carboxylic acid metabolic process [GO:0019752]	0.0007068	LYS21 LYS20	2	8
pyridine nucleotide biosynthetic process [GO:0019363]	0.0009058	BNA4 BNA1	2	9
methionine metabolic process [GO:0006555]	0.002253	MET3 MET16	2	14
negative regulation of fatty acid metabolic process [GO:0045922]	0.005149	FRM2	1	1
hydrogen sulfide biosynthetic process [GO:0070814]	0.005149	CYS4	1	1
cofactor biosynthetic process [GO:0051188]	0.005149	RNR4	1	1
cotranslational protein targeting to membrane [GO:0006613]	0.005149	HRI1	1	1
thiamine catabolic process [GO:0009230]	0.005149	THI20	1	1
sulfate assimilation, phosphoadenylyl sulfate reduction by phosphoadenylyl-sulfate reductase (thioredoxin) [GO:0019379]	0.005149	MET16	1	1
DNA fragmentation involved in apoptotic nuclear change [GO:0006309]	0.005149	YBL055C	1	1

S9 Fig. Highly expressed genes during micronutrient starvations compared with nitrogen starvations and genes specific to pantothenic acid and nicotinic acid starvation (cluster 14) during alcoholic fermentation.

For : N- **:** low nitrogen, 71 mg/L YAN; N-/Erg- : low nitrogen/low ergosterol, 71 mg/L YAN, 1.5 mg/L ergosterol; N+/Ole-: high nitrogen/ low oleic acid, 425 mg/L YAN, 18 mg/L oleic acid; N+/Erg-: high nitrogen/ low ergosterol, 425 mg/L YAN, 1.5 mg/L ergosterol; N+/Pan-: high nitrogen/ low pantothenic acid, 425 mg/L YAN, 0.02 mg/L pantothenic acid and N+/Nic-: high nitrogen/ low nicotinic acid, 425 mg/L YAN, 0.08 mg/L nicotinic acid; transcriptomic assays were performed at four time points during alcoholic fermentation (T1, 20 10^6 cells/mL; T2, 12 g CO_2 produced; T3, 40 g CO_2 produced; T4, 75 g CO_2 produced) indicated by (◢). Results show the mean of biological triplicate.
